# Risky sexual behaviors and associated factors among adolescent girls and young women in the context of HIV prevention pre-exposure prophylaxis in Tanzania: Analysis of the Tanzania HIV Impact Survey 2022–2023

**DOI:** 10.1371/journal.pgph.0007311

**Published:** 2026-10-23

**Authors:** Magreth Thadei Mwakilasa, Deodatus Kakoko, John Gilmore, Brynne Gilmore

**Affiliations:** 1 University College Dublin, School of Nursing, Midwifery and Health Systems, Dublin, Ireland; 2 Muhimbili University of Health and Allied Sciences, Dar es Salaam, United Republic of Tanzania; 3 UCD Centre for Interdisciplinary Research, Education and Innovation in Health Systems, University College Dublin, Dublin, Ireland; 4 Muhimbili University of Health and Allied Sciences, School of Public Health and Social Sciences, Dar es Salaam, United Republic of Tanzania; Babcock University School of Public and Allied Health, NIGERIA

## Abstract

Risky sexual behavior contributes to HIV acquisition among adolescent girls and young women (AGYW) in Sub-Saharan Africa (SSA), including Tanzania. However, nationally representative evidence on its prevalence and associated factors remains limited. This study examines the prevalence and associated factors of risky sexual behavior among AGYW aged 15–24 years in Tanzania, in the context of the expanding Pre-Exposure Prophylaxis (PrEP) provision. A secondary analysis of nationally representative data from the 2022–2023 Tanzania HIV Impact Survey (THIS) was conducted among 3,669 sexually active AGYW aged 15–24 who answered PrEP-related questions. Survey-weighted descriptive statistics, chi-square tests, and multivariable logistic regression were carried out. Adjusted odds ratios (AORs) with 95% confidence intervals (CIs) were reported, and statistical significance was set at p < 0.05.The survey-weighted prevalence of risky sexual behaviors was 38.29%, higher among those aged 15–17 years than 18–24 years (61.57% versus 35.63%). Not being in a union was associated with increased odds of risky sexual behaviors among participants aged 15–17 years (AOR = 8.72, 95% CI: 4.33-17.55; p < 0.001) and 18–24 years (AOR = 15.13, 95% CI: 11.40-20.08; p < 0.001). Alcohol use was also associated with increased odds in both age groups (15–17 years: AOR = 13.38, 95% CI: 1.53-116.64; p = 0.019; 18–24 years: AOR = 2.47, 95% CI: 1.57-3.89; p < 0.001). Among young women aged 18–24 years, health facility attendance, family planning use and willingness to use PrEP were associated with higher odds, whereas secondary or higher education, parent or guardian communication about sex or HIV, and prior HIV testing were associated with lower odds. Risky sexual behavior remains common among Tanzanian AGYW, particularly those aged 15–17 years. Age-responsive prevention programmes need to integrate behavioral and biomedical interventions, prioritizing AGYW who are not in a union and who use alcohol or have limited access to PrEP.

## Introduction

Risky sexual behaviors (RSBs) among adolescent girls and young women (AGYW) remain a major global public health concern, despite decades of biomedical, behavioral, and structural interventions to mitigate them [1–5]. RSBs in the context of HIV prevention are defined *“Any sexual behavior that increases the probability of exposure to HIV, including but not limited to unprotected genital contact (oral, anal, or vaginal), with or without penetration, orgasm, or ejaculation; concurrent sexual partners or multiple sequential sexual partners; sexual activity under the influence of drugs and/or alcohol; sexual activity in exchange for emotional support or material goods (e.g., money, drugs); and/or sexual activity with a partner of unknown HIV status*” [6]. The above indicators are also used by global, international, and national bodies to assess RSBs [7–9]. Sub-Saharan Africa (SSA) bears the highest HIV burden among AGYW, who are two to three times more likely to acquire HIV than their male counterparts [10].HIV incidence in SSA mirrors what is taking place in Tanzania, where adolescents and young people accounted for 34.3% of new HIV infections in 2022, with AGYW accounting for 74.1% [4,8,9]. Likewise, among AGYW, HIV prevalence is rising as they age from 0.8% among adolescent girls aged 15–19 years to 1.8% among young women aged 20–24 years [9]. While Tanzania has made notable progress in HIV treatment and testing coverage toward achieving the UNAIDS 95-95-95 targets, the new HIV incidence among AGYW remains high [8,9,11]. These incidences are occurring in the context of increasing availability of biomedical HIV prevention technologies, such as Pre-Exposure Prophylaxis (PrEP), alongside expanding PrEP choices and more comprehensive, integrated sexual and reproductive health (SRH) and HIV prevention services [7,10].

In SSA, AGYW are particularly vulnerable to HIV due to interacting biological, structural, and behavioral factors, including gender inequality, poverty, limited educational opportunities, unequal power in relationships, stigma surrounding sexual and reproductive health, and limited access to youth-friendly health services and information [5,12–17]. Together, these factors increase the likelihood of engaging in RSBs, including early sexual debut, inconsistent condom use, transactional sex, and age-disparate relationships [5,12–17]. These structural and behavioral issues are major contributors to the persistently high HIV incidence among AGYW, despite significant investments in prevention programmes across the region for a long time [5,13–15]. Therefore, the current study examines socio-demographic, behavioral, and PrEP-related factors and their associations with RSBs.

Empirical evidence from SSA consistently shows that RSB among AGYW are prevalent. A study carried out in Zambia shows that among sexually active female adolescents, 71.1% reported at least one RSB, including early sexual debut, non-use of condoms, transactional sex, alcohol use at last sex, or multiple partners [18]. In Uganda, 79.6% of sexually active AGYW reported inconsistent or no condom use, and 24.2% reported two or more sexual partners in the previous year, with out-of-school AGYW showing riskier profiles than those in school [19]. Previous studies conducted in Ethiopia, Rwanda, and South Africa link RSB to younger age, alcohol use, weak parental or social support, peer pressure, schooling disadvantage, residence, and community-level conditions [20–22]. Tanzania-specific evidence also shows that transactional sex and age-disparate partnerships are common among AGYW, reported at 35% and 28%, respectively, in a large prevention programme dataset [23,24]. Furthermore, a recent nationwide analysis found that 17.4% of female youth initiated sexual intercourse before age 18 years, with lower education strongly associated with early sexual debut [25,26]. Despite the global HIV prevention recommendations for comprehensive SRH services to reduce risk, which include HIV testing, consistent condom use, sexuality education, and PrEP for individuals at substantial risk of HIV infection, RSB persist across the SSA.

Despite growing evidence on RSB among AGYW, nationally representative evidence describing the overall prevalence of RSB and its associated socio-factors among Tanzanian AGYW aged 15–24 years in the context of PrEP availability remains limited. While the introduction of PrEP for HIV prevention has reshaped the sexual landscape around the world, particularly in terms of sexual behavior, practices, and preferences [27]. In the era of increasing PrEP use, research has raised concerns about actual or perceived behavioral risk compensation following PrEP initiation, with some studies suggesting that AGYW’s perceived protection from PrEP may influence engagement in RSB [28–30]; however, the evidence remains limited, particularly among AGYW.

Additionally, the reviewed literature on RSB was not conducted within the context of PrEP provision. Most previous studies focused on single behaviors or outcomes, such as early sexual initiation, HIV testing, HIV risk perception, or sexually transmitted infections (STIs) [24,26,31–35]. Furthermore, many were limited to specific population subgroups or local settings, including in-school adolescents, higher-education students, pregnant adolescents, rural adolescents, and program-enrolled adolescents [24,26,31–35]. Consequently, their findings may have limited generalizability to the broader population of AGYW eligible for PrEP services. Therefore, this study utilizes nationally representative data from the 2022–2023 Tanzania HIV Impact Survey (THIS**)** to explore the prevalence and factors associated with RSB among Tanzanian sexually active AGYW aged 15–24 years in the context of PrEP provision. Understanding the latter in the context of PrEP provision is essential for informing and designing equitable and evidence-based behavioral support services alongside HIV prevention and SRH programmes.

## Methods

### Study design

This study utilizes secondary data from the Population-based HIV Impact Assessment (PHIA), specifically the 2022/2023 THIS individual household based survey dataset [9]. PHIA surveys are cross-sectional, nationally representative, household-based surveys to produce population-level evidence on HIV, examine key HIV indicators, and assess the development and impact of national HIV response programs. The first-stage sampling units were defined by enumeration areas using a stratified multistage probability design; the second stage is defined by households within the enumeration areas; and the final stage is defined by age-related persons within the household [9]. The surveys include individuals aged 15 and above and use interviewer-administered questionnaires to collect comprehensive demographic, socio-economic, behavioral, and clinical data [9]. Furthermore, home-based HIV testing and counseling are conducted in line with national testing protocols, and laboratory confirmation and HIV viral load assessment are performed for individuals who test positive for HIV. Data collection for 2022–2033 was conducted from November 2022 to March 2023. More detailed information on the THIS survey design, including sampling, recruitment, and data collection procedures, has been published elsewhere [9]. Permission to access the dataset for this analysis was granted after submission on October 17, 2024, and approval on October 24, 2024. Also, because the current study relies on secondary data, the researchers had no direct contact with participants and were unable to identify them in the survey.

### Data access and ethical approval

The study used secondary de-identified data to answer the research questions. Access to utilize the dataset for this analysis was obtained in October 2025 from the PHIA-ICAP Columbia University website after submitting a data analysis plan. The PHIA surveys received ethical approval from international and local human subjects institutional review boards, including the U.S. Centers for Disease Control and Prevention (CDC), and national ethics committees such as the National Institute for Medical Research (NIMR) in Tanzania (reference number NIMR/HQ/R.8a/to/4125). The institutional review boards at ICAP at Columbia University or the University of Maryland, Baltimore. The PHIA surveys were funded by the U.S. President’s Emergency Plan for AIDS Relief (PEPFAR), with technical assistance provided by the CDC under cooperative agreements #U2GGH002173 and #U2GGH002172.In the original PHIA surveys, all participants provided written informed consent before participation; for minors aged 15–17 years, written assent was obtained in addition to parental or guardian permission. No further ethical approval was obtained from local review committees in Tanzania for reanalyzing this secondary data, aside from the approval granted to access and use the data from PHIA.

### Study population

To answer the research question, this study included data from participants in the PHIA-THIS 2022/2023 dataset who met the following criteria: were 15–24 years old, were female, tested negative for HIV, reported being sexually active, had a missing value for the outcome composite variable, and responded to all three survey questions on PrEP awareness, willingness, and use. Data from respondents who did not meet all these criteria were excluded. The recent survey included 35,957 respondents aged 15 years and older. After applying the inclusion criteria, a sample size of 3669 sexually active AGYW was obtained. See Fig 1 below for more details.

**Fig 1:**
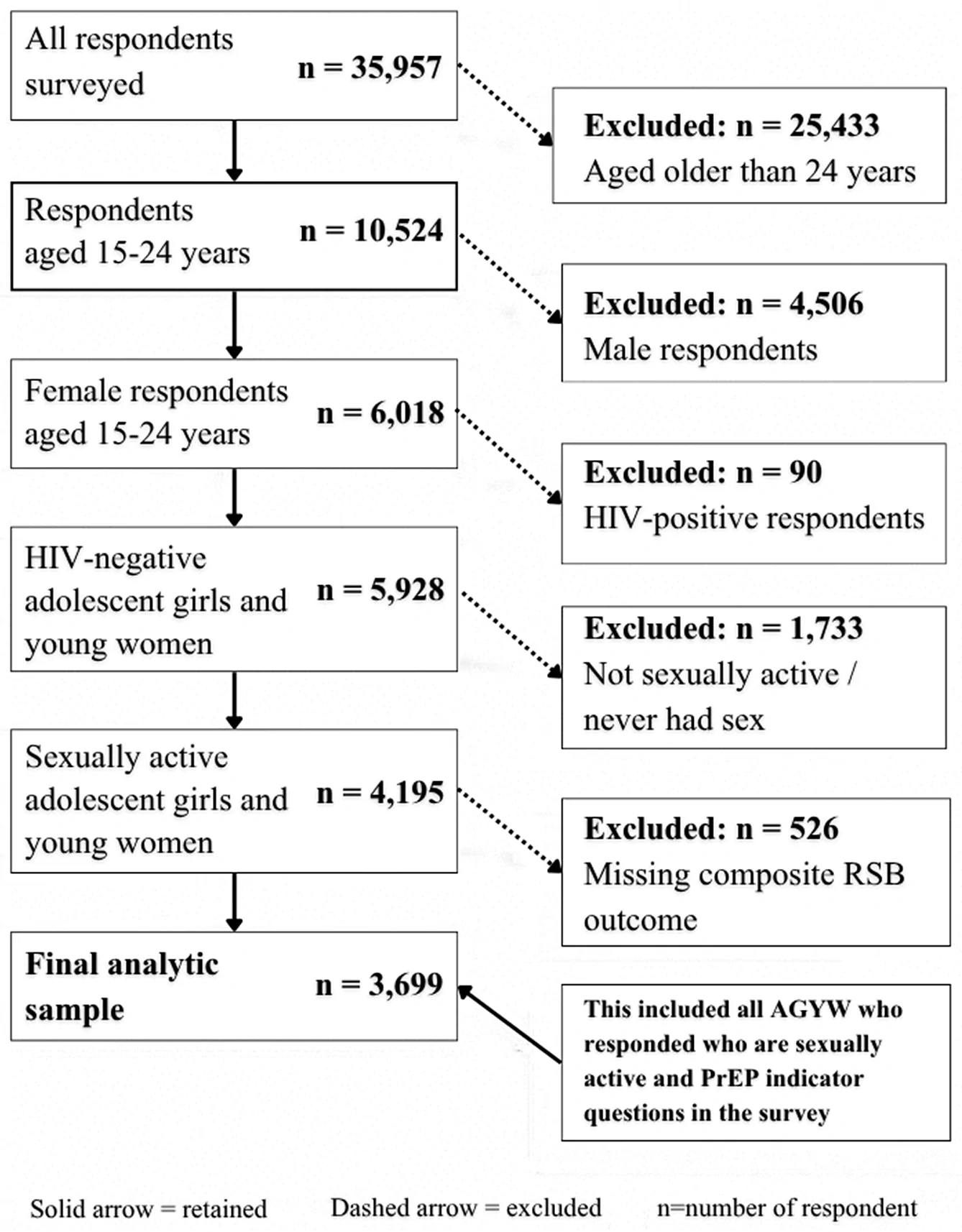
Participant selection flow for adolescent girls and young women included in the Tanzania HIV Impact Survey 2022-2023.

### Variable of measurement

#### Dependent variable.

The dependent variable was the composite RSB, treated as a binary variable. Respondents were considered engaging in RSB if they reported any of the following as coded as “Yes”. (1) sexual debut before the age of 15 years; (2) having multiple sexual partners in the previous 12 months; and (3) engaging in sexual intercourse without a condom with a non-marital or non-cohabiting partner in the past 12 months. Respondents were coded “No” if they did not have RSB only when all component variables were observed and none of the behaviors were present, or when they were structurally missing. Respondents with true missing information on any of the included variables were excluded from the final analysis, S1 Table. Additionally, other important indicators of RSB, such as inconsistent condom use, transactional sex, sex under the influence of substances, and age-disparate relationships, were not included in the making of the composite variables because they were not captured in the survey.

#### Independent variables.

The independent variables of interest included a range of socio-demographic, behavioral, and PrEP-related factors. These included age (15–17/18–24), educational attainment (no education/Primary/secondary+), employment status in the past 12 months, place of residence (urban, rural), union status (in a union or not in a union), age at first marriage or cohabiting below 15, yes; above, no; and missingness was treated as no and wealth status (poor/middle/rich). The geographical zone was a derived variable based on participants’ regions of residence. The 31 regions of Tanzania were grouped into eight geographical zones: Eastern (Dar es Salaam, Morogoro, and Pwani), Northern (Arusha, Kilimanjaro, Manyara, and Tanga), Lake (Geita, Kagera, Mara, Mwanza, Shinyanga, and Simiyu), Western (Katavi, Kigoma, and Tabora), Central (Dodoma and Singida), Southern Highlands (Iringa, Mbeya, Njombe, Rukwa, and Songwe), Southern (Lindi, Mtwara, and Ruvuma), and Zanzibar (Pemba North, Pemba South, Unguja North, Unguja South, and Mjini Magharibi).

Behavioral variables included access to a health facility in the past 12 months, current family planning use, ever testing for HIV, and condom use at the last sexual intercourse in the past 12 months, all measured as binary variables (yes/no). Discussion about sex or HIV with a parent or guardian was assessed using two separate binary variables, indicating yes or no for each topic. Additionally, recent alcohol use was evaluated with two variables: alcohol consumption in the past 12 months and alcohol use in the past 3 months, both recorded as yes or no. PrEP-related indicators included PrEP awareness, willingness, and use, measured as binary (yes/no). During each PHIA survey, PrEP was described as a daily medication taken to prevent HIV infection before potential exposure. All respondents who reported being HIV-negative were asked whether they had previously heard of PrEP. Those who were aware were asked whether they had ever used PrEP, and those who had never used it, including respondents introduced to PrEP during the interview, were asked about their willingness to use it for HIV prevention. See Fig 2 below for more details.

**Fig 2:**
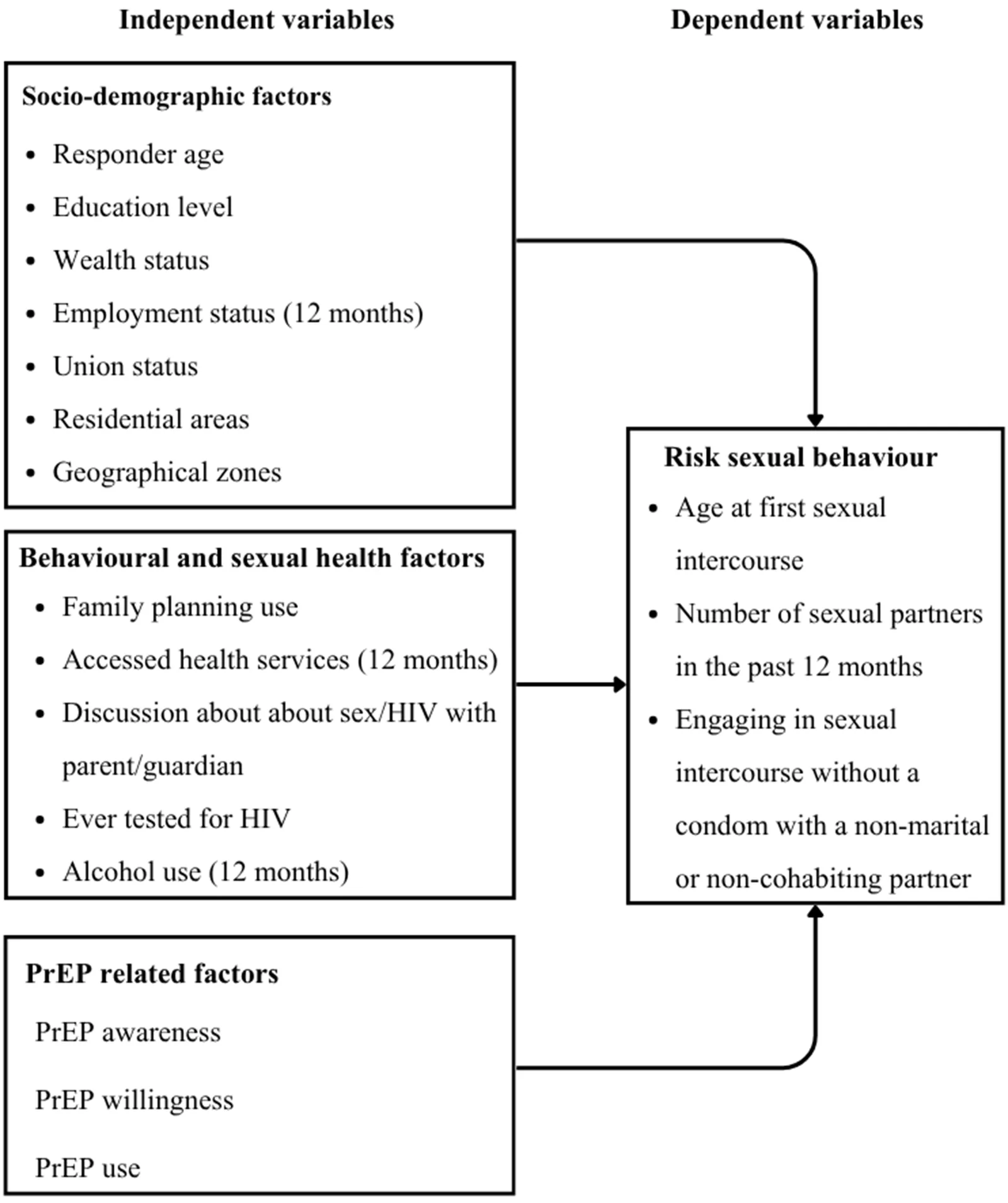
A conceptual framework of factors associated with risky sexual behaviors among adloescent girls and young women.

### Statistical analysis

Data analysis was conducted using Stata version 18.0, accounting for the complex survey design using *“svyset”* and “*svy*” commands, incorporating sampling weights, stratification, primary sampling units, and jackknife variance-estimated replicates to obtain nationally representative estimates and valid standard errors. The analyses accounted for a two-stage stratified cluster sampling design and used the survey-provided weights and replicated weights for variance estimation at the PSU level. Missing values were handled using the available-cases approach in the multivariate model, in which respondents with missing data on the variables in each analysis were excluded S1 Fig. However, in the descriptive analyses, missing values were left as is to allow readers to understand the amount of missing data for each variable.

Descriptive statistics were computed for the weighted samples to summarize respondent characteristics and the prevalence of RSB. Chi-square tests were subsequently performed to examine associations between independent factors and RSB. Grouped bar graphs were used to present the prevalence of RSB. Crude odds ratios (CORs) and adjusted odds ratios (AORs), with corresponding 95% confidence intervals (CIs), were estimated using univariate and multivariate logistic regression models to identify factors associated with RSB. Two models were run: one for girls aged 15–17 and the second for those aged 18–24 years, and the *subpopulation* was added to the common age stratification. Statistical significance was determined using a p-value threshold of <0.05. Multicollinearity among the predictors was assessed separately for each age group using variance inflation factors (VIFs) and tolerance values. Among adolescent girls aged 15–17 years, VIFs ranged from 1.03 to 2.86, with a mean of 1.51, while among young women aged 18–24 years, VIFs ranged from 1.02 to 3.53, with a mean of 1.53. As all VIFs were below 5 and all tolerance values exceeded 0.20, there was no evidence of problematic multicollinearity in either multivariable model. Model goodness-of-fit was assessed using the Hosmer-Lemeshow test (*estat gof*). Additionally, sensitivity analysis included marriage or cohabitation before age 15 as one indicator of the composite RSB. The RSB composite and the survey-weighted regression model were repeated to assess the robustness of the findings, S2 Table. The study was reported in accordance with the Strengthening the Reporting of Observational Studies in Epidemiology (STROBE) guideline for cross-sectional quantitative studies, see S1 File.

## Results

Overall, 3669 AGYW with sexual activity were included in this analysis; S1 Fig for analytical sample selection. In the study, most participants were aged 18–24 years (89.75%), had primary education (53.28%), had worked in the previous 12 months (65.50%), and lived in rural areas (61.71%). Among adolescent girls aged 15–17 years, RSB was significantly associated only with union status (F (1, 173) = 54.19, *p* < 0.001), marriage before 15 years F (1, 173) = 11.21, *p* = 0.001), family planning (F (1.99, 344.33) = 5.15, *p* = 0.006), and condom use at last sexual intercourse (F (1.98, 341.76) = 21.82, *p* < 0.001). Among young women aged 18–24 years, all factors had significant associations with risk sexual behavior, except for discussion of sex/HIV (F(1, 271) = 0.92, *p* = 0.337), PrEP awareness (F(1, 271) = 0.25, *p* = 0.620), or PrEP use (F(1, 271) = 0.21, *p* = 0.646). See Table 1 below for more details.

**Table 1:** Weighted sociodemographic, behavioral, and HIV prevention characteristics and their association with risky sexual behavior among adolescent girls and young women, and bivariate associations stratified by age group in the THIS 2022-2023 survey.

Participant characteristic	Overall N = 3,669 n (weighted %)	Adolescent girls 15–17 years n=351 (10.25%)			Young women 18–24 years n=3,298 (89.75%)		
		No RSB n (weighted row %)	Yes RSB n (weighted row %)	p-value	No RSB n (weighted row %)	Yes RSB n (weighted row %)	p-value
**Education**				0.414			<0.001*
No education	398 (10.17)	21 (47.18)	22 (52.82)		246 (67.55)	109 (32.45)	
Primary	2,004 (53.28)	93 (38.72)	136 (61.28)		1,243 (67.60)	532 (32.40)	
Secondary and above	1,267 (36.55)	27 (33.87)	53 (66.13)		724 (59.09)	463 (40.91)	
**Worked in the previous 12 months**				0.381			0.028*
No	1,280 (34.50)	34 (33.75)	55 (66.25)		747 (61.43)	444 (38.57)	
Yes	2,389 (65.50)	107 (40.08)	156 (59.92)		1,466 (65.98)	660 (34.02)	
**Wealth index**				0.140			<0.001*
Poor	1,676 (43.49)	87 (40.58)	120 (59.42)		1,052 (69.65)	417 (30.35)	
Middle	739 (18.98)	35 (43.97)	39 (56.03)		435 (64.13)	230 (35.87)	
Rich	1,254 (37.53)	19 (27.60)	52 (72.40)		726 (58.86)	457 (41.14)	
**Residential location**				0.127			<0.001*
Urban	1,281 (38.29)	28 (31.35)	62 (68.65)		713 (57.43)	478 (42.57)	
Rural	2,388 (61.71)	113 (41.14)	149 (58.86)		1,500 (68.90)	626 (31.10)	
**Geographical zone**				0.390			<0.001*
Central	362 (8.91)	13 (38.33)	19 (61.67)		231 (69.34)	99 (30.66)	
Northern	182 (9.27)	2 (14.77)	10 (85.23)		103 (60.66)	67 (39.34)	
Western	278 (9.62)	11 (37.08)	20 (62.92)		161 (66.88)	86 (33.12)	
Lake Zone	1,105 (30.79)	60 (40.46)	79 (59.54)		667 (67.37)	299 (32.63)	
Southern Highlands	386 (5.32)	6 (28.80)	22 (71.20)		223 (63.66)	135 (36.34)	
Southern	112 (3.75)	4 (31.54)	7 (68.46)		46 (43.79)	55 (56.21)	
Eastern	597 (19.91)	19 (39.21)	27 (60.79)		333 (56.62)	218 (43.38)	
South-West Highlands	525 (10.36)	24 (50.40)	24 (49.60)		348 (72.07)	129 (27.93)	
Zanzibar	122 (2.08)	2 (44.47)	3 (55.53)		101 (82.35)	16 (17.65)	
**Union status**				<0.001*			<0.001*
In union	2,625 (68.53)	95 (65.45)	49 (34.55)		2,014 (80.29)	467 (19.71)	
Not in union	1,043 (31.45)	46 (21.13)	162 (78.87)		198 (23.56)	637 (76.44)	
**Age at marriage/cohabitation before age 15**				0.001*			<0.001*
No	3,555 (97.17)	140 (40.14)	192 (59.86)		2,205 (65.88)	1,018 (34.12)	
Yes	113 (2.81)	1 (4.50)	19 (95.50)		7 (6.93)	86 (93.07)	
Missing	1 (0.02)	–	–		1 (100.00)	0 (0.00)	
**Visited a health facility in the previous 12 months**				0.075			<0.001*
No	2,148 (58.13)	67 (43.37)	87 (56.63)		1,391 (67.86)	603 (32.14)	
Yes	1,521 (41.87)	74 (34.54)	124 (65.46)		822 (59.18)	501 (40.82)	
**Discussed sex/HIV with parents or guardians**				0.953			0.292
No	2,936 (78.39)	119 (38.35)	176 (61.65)		1,750 (63.83)	891 (36.17)	
Yes	733 (21.61)	22 (38.80)	35 (61.20)		463 (66.28)	213 (33.72)	
**Currently using a family-planning method**				0.008*			<0.001*
No	1,043 (26.90)	20 (42.17)	23 (57.83)		732 (71.79)	268 (28.21)	
Yes	2,039 (57.43)	91 (33.75)	165 (66.25)		1,085 (58.49)	698 (41.51)	
Missing	587 (15.66)	30 (58.11)	23 (41.89)		396 (71.71)	138 (28.29)	
**Ever tested for HIV**				0.353			<0.001*
No	473 (13.61)	60 (35.77)	100 (64.23)		134 (37.90)	179 (62.10)	
Yes	3,196 (86.39)	81 (40.60)	111 (59.40)		2,079 (67.32)	925 (32.68)	
**Alcohol use**				0.182			<0.001*
No	3,417 (93.37)	138 (39.40)	199 (60.60)		2,091 (65.51)	989 (34.49)	
Yes	252 (6.63)	3 (18.73)	12 (81.27)		122 (48.86)	115 (51.14)	
**Condom use at last sexual encounter in the previous 12 months**				<0.001*			<0.001*
Used condom	374 (10.55)	41 (77.10)	11 (22.90)		273 (85.11)	49 (14.89)	
Did not use condom	3,256 (88.44)	100 (33.75)	176 (66.25)		1,938 (62.29)	1,042 (37.71)	
No sex in previous 12 months	33 (0.86)	0 (0.00)	24 (100.00)		0 (0.00)	9 (100.00)	
Missing	6 (0.14)	–	–		2 (43.91)	4 (56.09)	
**PrEP awareness**				0.419			0.690
No	3,459 (94.22)	132 (37.89)	201 (62.11)		2,092 (64.46)	1,034 (35.54)	
Yes	210 (5.78)	9 (47.24)	10 (52.76)		121 (62.87)	70 (37.13)	
**PrEP willingness**				0.687			0.001*
No	2,334 (62.96)	94 (39.40)	132 (60.60)		1,462 (67.14)	646 (32.86)	
Yes	1,335 (37.04)	47 (36.71)	79 (63.29)		751 (59.68)	458 (40.32)	
**PrEP use**				0.465			0.619
No	3,640 (99.13)	140 (38.26)	210 (61.74)		2,195 (64.33)	1,095 (35.67)	
Yes	29 (0.87)	1 (63.45)	1 (36.55)		18 (69.12)	9 (30.88)	

**Abbreviations:** RSB = Risky sexual behavior; PrEP = pre-exposure prophylaxis, adjusted F statistics and P-values are from the survey-adjusted Pearson χ^2^ test, Statistical significance was defined as P < 0.05, N = Number of respondents, (%) = Percentages.

### Prevalence of risky sexual behavior among adolescent girls and young women in Tanzania

Fig 3 below shows the overall survey-weighted prevalence of risky sexual behavior: 38.29% (95% CI: 36.32-40.29) of AGYW reported engaging in RSB. The prevalence was higher among adolescent girls aged 15–17 years (61.57%, 95% CI: 56.27-66.62) than among young women aged 18–24 years (35.63%, 95% CI: 33.67-37.64). Early sexual debut at age 14 years or younger was also more common among adolescent girls aged 15–17 years (25.80%, 95% CI: 21.37-30.79) than among young women (7.41%, 95% CI: 6.43-8.53). Condomless sex with a non-marital/non cohabiting partner followed the latter pattern, 61.91% versus 36.17%, respectively. In contrast, the prevalence of reporting two or more sexual partners in the past 12 months was similar in both age groups, at approximately 7%. Further details are presented in S3 Table.

**Fig 3:**
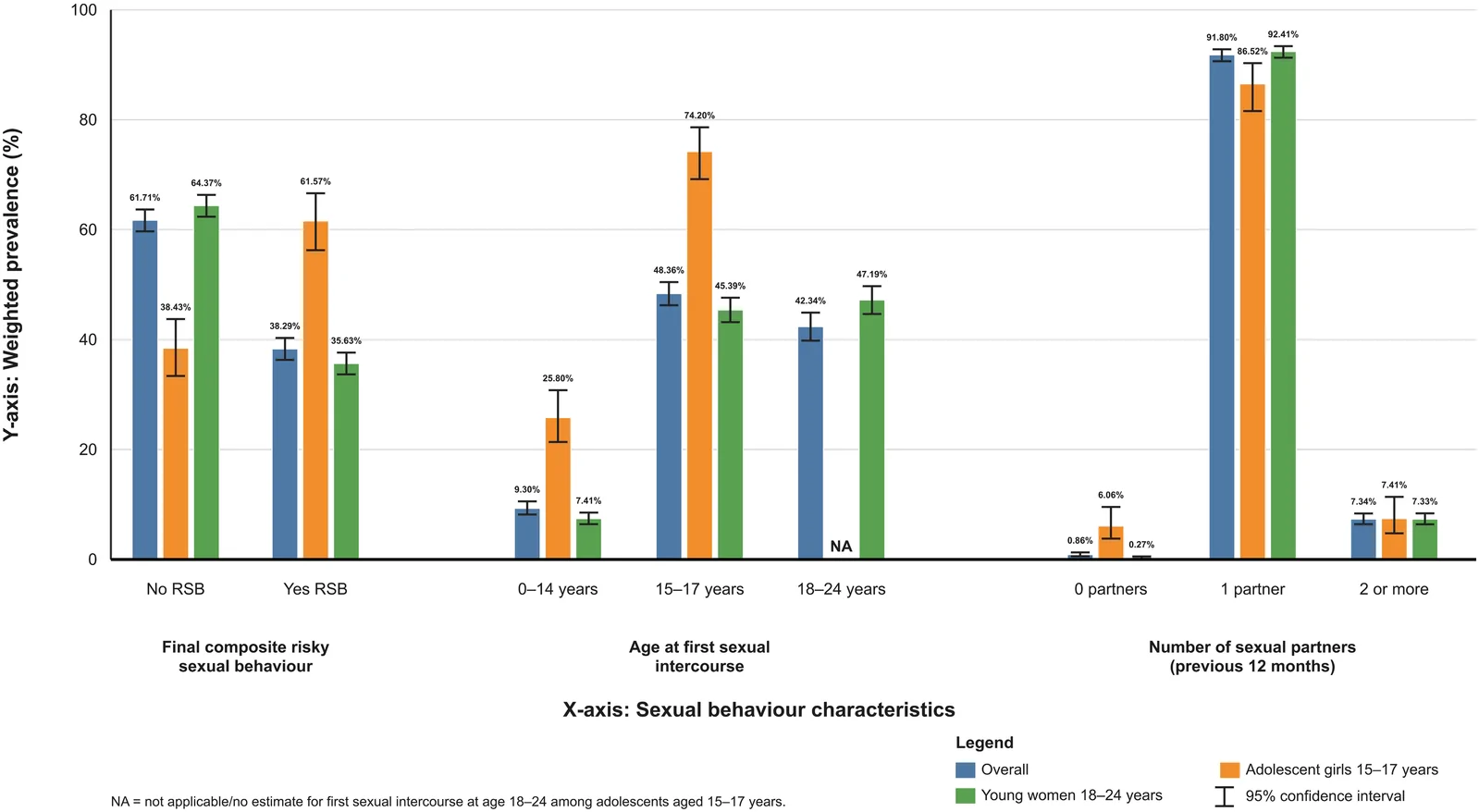
Survey-weighted prevalence of risky sexual behaviour and selected sexual behaviour characteristics by age group.

### Factors associated with risky sexual behavior among AGYW in Tanzania

Table 2 shows that in a multivariate model, only not being in a union (AOR = 8.72, 95% CI: 4.33-17.55; p < 0.001) and alcohol use (AOR = 13.38, 95% CI: 1.53-116.64; p = 0.019) were associated with higher odds of RSB among adolescent girls aged 15–17 years. Among young women aged 18–24 years, higher odds of RSB were associated with not being in a union (AOR = 15.13, 95% CI: 11.40-20.08; p < 0.001), visiting a health facility in the previous 12 months (AOR = 1.29, 95% CI: 1.03-1.60; p = 0.025), currently using family planning (AOR = 1.31, 95% CI: 1.03-1.68; p = 0.030), alcohol use (AOR = 2.47, 95% CI: 1.57-3.89; p < 0.001) and PrEP willingness (AOR = 1.37, 95% CI: 1.05-1.78; p = 0.020). In contrast, secondary or higher education (AOR = 0.55, 95% CI: 0.38-0.82; p = 0.003), discussing sex or HIV with parents or guardians (AOR = 0.60, 95% CI: 0.45-0.79; p < 0.001) and ever testing for HIV (AOR = 0.61, 95% CI: 0.44-0.85; p = 0.003) were associated with lower odds of RSB among young women.. Employment, wealth, residential location, and PrEP awareness were not significantly associated with RSB in either age group. Both adjusted models were statistically significant overall, and the goodness-of-fit tests provided no evidence of poor fit.

**Table 2:** Survey-weighted multivariate logistic regression models exploring factors associated with risky sexual behavior among AGYW in the THIS survey.

Variable and categories	Adolescent girls aged 15–17 years				Young women aged 18–24 years			
	COR (95% CI)	p-value	AOR (95% CI)	p-value	COR (95% CI)	p-value	AOR (95% CI)	p-value
**Education**								
No education	*1.00 (Ref.)*	—	*1.00 (Ref.)*	—	*1.00 (Ref.)*	—	*1.00 (Ref.)*	—
Primary	1.41 (0.68-2.95)	0.353	1.23 (0.53-2.86)	0.633	1.00 (0.77-1.30)	0.987	0.81 (0.56-1.16)	0.242
Secondary and beyond	1.74 (0.80-3.81)	0.161	0.94 (0.34-2.59)	0.902	1.44 (1.12-1.85)	0.004*	0.55 (0.38-0.82)	0.003*
**Worked in previous 12 months**								
No	*1.00 (Ref.)*	—	*1.00 (Ref.)*	—	*1.00 (Ref.)*	—	*1.00 (Ref.)*	—
Yes	0.76 (0.41-1.41)	0.382	0.73 (0.34-1.53)	0.399	0.82 (0.69-0.98)	0.028*	0.81 (0.64-1.03)	0.086
**Wealth index**								
Poor	*1.00 (Ref.)*	—	*1.00 (Ref.)*	—	*1.00 (Ref.)*	—	*1.00 (Ref.)*	—
Middle	0.87 (0.47-1.60)	0.654	0.47 (0.20-1.11)	0.085	1.28 (1.02-1.62)	0.035*	0.95 (0.69-1.31)	0.761
Rich	1.79 (0.90-3.56)	0.096	0.69 (0.23-2.06)	0.499	1.60 (1.31-1.96)	<0.001*	0.89 (0.64-1.25)	0.510
**Residential location**								
Urban	*1.00 (Ref.)*	—	*1.00 (Ref.)*	—	*1.00 (Ref.)*	—	*1.00 (Ref.)*	—
Rural	0.65 (0.38-1.13)	0.128	0.87 (0.35-2.17)	0.764	0.61 (0.51-0.72)	<0.001*	0.84 (0.65-1.08)	0.172
**Union status**								
In union	*1.00 (Ref.)*	—	*1.00 (Ref.)*	—	*1.00 (Ref.)*	—	*1.00 (Ref.)*	—
Not in union	7.07 (4.09-12.21)	<0.001*	8.72 (4.33-17.55)	<0.001*	13.22 (10.51-16.63)	<0.001*	15.13 (11.40-20.08)	<0.001*
**Visited a health facility in the previous 12 months**								
No	*1.00 (Ref.)*	—	*1.00 (Ref.)*	—	*1.00 (Ref.)*	—	*1.00 (Ref.)*	—
Yes	1.45 (0.96-2.19)	0.076	1.29 (0.67-2.50)	0.444	1.46 (1.24-1.72)	<0.001*	1.29 (1.03-1.60)	0.025*
**Discussed sex/HIV with parents or guardians**								
No	*1.00 (Ref.)*	—	*1.00 (Ref.)*	—	*1.00 (Ref.)*	—	*1.00 (Ref.)*	—
Yes	0.98 (0.51-1.87)	0.953	0.70 (0.27-1.87)	0.480	0.90 (0.73–1.10)	0.293	0.60 (0.45-0.79)	<0.001*
**Currently using family planning**								
No	*1.00 (Ref.)*	—	*1.00 (Ref.)*	—	*1.00 (Ref.)*	—	*1.00 (Ref.)*	—
Yes	1.43 (0.70-2.91)	0.319	1.68 (0.71-3.97)	0.237	1.81 (1.49-2.20)	<0.001*	1.31 (1.03-1.68)	0.030*
**Alcohol use**								
No	*1.00 (Ref.)*	—	*1.00 (Ref.)*	—	*1.00 (Ref.)*	—	*1.00 (Ref.)*	—
Yes	2.82 (0.57-13.86)	0.200	13.38 (1.53-116.64)	0.019*	1.99 (1.44-2.74)	<0.001*	2.47 (1.57-3.89)	<0.001*
**Ever tested for HIV**								
No	*1.00 (Ref.)*	—	*1.00 (Ref.)*	—	*1.00 (Ref.)*	—	*1.00 (Ref.)*	—
Yes	0.81 (0.53-1.26)	0.353	1.52 (0.80-2.89)	0.198	0.30 (0.23-0.37)	<0.001*	0.61 (0.44-0.85)	0.003*
**PrEP awareness**								
No	*1.00 (Ref.)*	—	*1.00 (Ref.)*	—	*1.00 (Ref.)*	—	*1.00 (Ref.)*	—
Yes	0.68 (0.27-1.75)	0.422	0.54 (0.18-1.63)	0.275	1.07 (0.76-1.50)	0.690	0.93 (0.58-1.51)	0.777
**PrEP willingness**								
No	*1.00 (Ref.)*	—	*1.00 (Ref.)*	—	*1.00 (Ref.)*	—	*1.00 (Ref.)*	—
Yes	1.12 (0.64-1.96)	0.687	1.01 (0.57-1.78)	0.970	1.38 (1.14-1.67)	0.001*	1.37 (1.05-1.78)	0.020*

**Model statistics.** Ages 15–17 years (adjusted subpopulation n = 299): F (14, 151) = 3.80, p < 0.001; goodness-of-fit F (9, 29) = 0.47, p = 0.881. Ages 18–24 years (adjusted subpopulation n = 2,782): F (14, 252) = 36.57, p < 0.001; goodness-of-fit F (9, 256) = 1.30, p = 0.239.

**Notes.** COR = crude odds ratio; AOR = adjusted odds ratio; CI = confidence interval; PrEP = pre-exposure prophylaxis; RSB = risky sexual behavior; Ref. = reference category. *p < 0.05. CORs are from separate univariable survey subpopulation models; AORs are mutually adjusted survey subpopulation estimates for all variables shown. The goodness-of-fit statistics were obtained from age-restricted survey logistic fits because Stata does not allow *estat gof* after subpop () estimation.

## Discussion

This study used nationally representative data from the PHIA-THIS 2022/202 to estimate the prevalence of RSB and associated factors among sexually active AGYW in the era of PrEP provision. The overall weighted prevalence of RSB was 38.29%, with a substantially higher prevalence among adolescent girls aged 15–17 years than among young women aged 18–24 years. Not being in union and alcohol use were associated with higher odds of RSB in both age groups. Among young women aged 18–24 years, visiting a health facility, using family planning, and willingness to use PrEP were associated with higher odds of RSB, whereas secondary or higher education, discussion of sex/HIV with parents or guardians, and previous HIV testing were associated with lower odds.

This observed prevalence confirms that RSB remains an important SRH concern among Tanzanian AGYW and is consistent with evidence from other SSA countries with high burden of HIV and STIs incidence [35]. The prevalence of RSB in this study was lower than that reported in Zambia (71.1%) and Rwanda (41%) but higher than the school-based prevalence from Ethiopia (17.2%) [18,20,21]. Differences between studies may reflect variations in study populations, settings, age ranges, and the definition of RSB adopted in prior studies. The particularly high prevalence among adolescent girls aged 15–17 years in the era of PrEP availability is concerning, because sexual initiation commonly occurs during this developmental period in Tanzania [23,24,26]. Acknowledging that both risk and health-related behaviors develop during these stages, shaping behavior and health over the life course. These findings suggest that Tanzanian AGYW share the wider regional pattern of persistent sexual vulnerability among AGYW, and SRH education should be provided before, during, and after sexual initiation.

Not being in a union was strongly associated with high RSB across both age groups, consistent with evidence from Zambia, Ethiopia, and Rwanda [17,19,20,36]. AGYW who were not in a union may experience greater relationship transitions and sexual autonomy, and may be more likely to have non-regular or multiple sexual partners, inconsistent condom use, and engage in transactional sex [18,21,23,32]. However, union status should not be interpreted as inherently protective, as risk also depends on partner characteristics, relationship power, and condom use. This finding underscores the need for early SRH education that equips unmarried AGYW with the knowledge and skills to make informed sexual and relationship decisions. Alcohol use was similarly associated with higher odds of RSB in both age groups. Prior studies have linked alcohol consumption with condomless or unprotected sex, multiple sexual partnerships, and impaired sexual decision-making [20,21,24,36–40]. Alcohol may reduce the ability to assess risk or negotiate safer sex and may occur in social environments where other RSB are common. Additionally, the particularly large confidence interval among adolescent girls aged 15–17 years may indicate substantial statistical imprecision; therefore, it should be interpreted cautiously. The latter can be a result of a relatively small number of sexually active participants and alcohol users in the adolescent girls model. Despite the observed imprecision, the current finding underscores that alcohol use is a driver of RSB among AGYW and supports integrating continuous alcohol risk screening and addressing alcohol consumption alongside HIV or SRH prevention efforts in Tanzania.

Among young women aged 18–24 years who visited a health facility in the previous 12 months and were using family planning, higher odds of RSB were observed, consistent with prior evidence [37,41–43]. However, this finding should not be interpreted as evidence that health services or contraceptive use cause RSB. Instead, they may reflect risk-related service uptake, whereby sexually active young women or those concerned about pregnancy, STIs, or HIV exposure are more likely to seek care [37,41]. Previous studies have similarly shown that contraception users may report frequent sexual activity while recognizing their continued risk of unintended pregnancy and STIs [42,43]. Qualitative studies further showed that young women, in some cases, prioritize pregnancy prevention over HIV prevention, and that condoms and contraception are not always used for dual protection [44–46]. Health facility and family planning visits therefore represent an opportunity to provide integrated HIV/STIs prevention, including risk assessment, condom provision, HIV testing, PrEP information, and referrals, consistent with WHO guidance [47]. The current finding indicates a missed opportunity to strengthen comprehensive HIV prevention and SRH health services in the country, especially within healthcare facilities, when AGYW visit for family planning or seek other services. The current findings imply that access to the healthcare facility is an endpoint and that young women receive only the services they seek. Also, the lack of association between visiting a health facility and family planning use and RSB among adolescent girls aged 15–17 years may be due to variation in study populations and sizes, an age-stratified modelling approach comparing factors, and a focus on sexually active AGYW who responded to PrEP-related indicator questions in the survey

Secondary or higher education, communication about sex/HIV with a parent or guardian, and prior HIV testing were associated with lower odds of RSB among young women aged 18–24 years, although these associations were not observed among adolescent girls aged 15–17 years. The current finding suggests that education may strengthen SRH knowledge, decision-making, and safer sex negotiation while supporting delayed sexual initiation, consistent with previous research [35,48–50]. These findings support efforts to retain girls in secondary education and beyond and to provide targeted HIV prevention and SRH services to those who are out of school or have limited educational opportunities. Parent or guardian communication may also provide guidance, raise awareness, delay sexual debut, and inform sexual-related decisions. Prior studies from Tanzania showed that communication about sexuality is limited at the family and community levels and is often constrained by cultural norms, embarrassment, or fear-based or warning-based messaging, so that AGYW do not learn anything in the process [51,52]. Peers and schools consequently become important sources of SRH information. However, the available school curriculum is often insufficient to address AGYW questions, leading many to rely on inaccurate or incomplete information from peers and other informal sources. [53]. The study emphasizes that family-centered intervention should therefore help parents and guardians communicate accurately, respectfully, and without judgment.

HIV testing was not associated with lower odds of RSB among young women aged 18–24, but not among adolescent girls aged 15–17 years. The current findings suggest that accessing HIV testing may reduce risk when accompanied by high-quality counseling, linkage to prevention services, and continued support. However, findings across SSA are mixed, with some studies reporting lower odds, others higher odds, and others finding no association with HIV testing [37,54–58]. These differences may arise because some individuals seek testing after recognizing a recent exposure, whereas others receive testing as part of ongoing preventive care. The current study therefore recommends that HIV testing should be treated as an entry point for comprehensive prevention rather than a stand-alone intervention.

PrEP awareness, willingness, and current PrEP use were low in Tanzania, consistent with prior studies [59–62]. PrEP awareness was not associated with RSB in either age group, whereas willingness to use PrEP was associated with higher odds of RSB among young women aged 18–24 years and was not significant among adolescent girls aged 15–17 years. This association is likely to indicate that PrEP willingness encourages RSB; instead, it may reflect accurate recognition of HIV vulnerability among young women experiencing condomless sex, multiple partners, partner HIV risk, and age-disparate partnerships [41,63,64]. In accordance with the health belief model, greater perceived susceptibility may increase willingness to adopt PrEP. Therefore, expanding youth-friendly PrEP access and continuation support is essential, alongside qualitative and longitudinal research to understand the relationship between perceived risk, willingness, and actual uptake.

### Limitations, strengths and future direction

A major strength is that it used nationally representative data from the THIS 2022–2023, making the findings generalizable to sexually active adolescent girls and young women in Tanzania. To our knowledge, this is the first nationally representative study in Tanzania to examine RSB in the context of PrEP availability, thereby providing important evidence for HIV prevention policy and programme implementation. However, the cross-sectional design limits causal inference and prevents determination of temporal order between RSB and associated factors. Second, RSB indicators were self-reported and involved sensitive behaviors, so social desirability and recall bias may have led to underreporting of condomless sex, alcohol use, and other stigmatized practices. Third, comparisons across studies should be interpreted cautiously because RSB definitions vary widely by setting, with some studies including early debut, transactional sex, or condom use constructs differently.

Finally, the current study adopted the traditional definition of RSB, which is commonly used in HIV prevention research [6]. The process of developing the composite variable revealed limitations in existing definitions and highlighted the need to reconceptualize RSB in the era of effective biomedical HIV prevention. The traditional definitions of RSB were developed before the rapid availability of antiretroviral therapy for HIV prevention, such as PrEP, when condomless sex was directly linked to HIV acquisition risk [6]. But as PrEP and treatment as prevention (U = U) become more widely available, the relationship between sexual behavior and HIV risk is a more complex one. For people who are adherent to PrEP or whose partner has sustained viral load suppression, the risk of acquiring HIV may not be as great with condomless sex, but it is still important for preventing STIs, unintended pregnancy, and other adverse SRH outcomes.

Consequently, there is growing recognition that traditional definitions of RSB may no longer adequately capture HIV risk and should be reconceptualized to reflect the contemporary prevention landscape. Instead of looking at sexual behavior as inherently risky, we need to look at risk in the wider context of biomedical protection, adherence to PrEP, partner viral suppression, relationships, reproductive intentions, and access to integrated SRH services. Future research should therefore develop and validate context-specific measures of sexual risk that reflect the realities of the PrEP era, especially among women at high risk of HIV acquisition.

## Conclusion

Risky sexual behavior remains common among Tanzanian adolescent girls and young women, particularly among those aged 15–17 years. Not being in a union and alcohol were associated with increased odds of risky sexual behavior in both age groups. Among young women aged18–24 years, access to health care facilities, family planning, communication, education, HIV testing, and willingness to use PrEP were associated with RSB. Therefore, these findings support early access to age-responsive, comprehensive SRH education programs that integrate HIV prevention, alcohol risk reduction, parent or guardian communication, HIV testing, family planning services, and accessible PrEP counseling. Such programmes can enable AGYW to make informed choices about their sexual relationships and health, with particular attention paid to those who are not in a union and use alcohol.

## Supporting information

S1 Table Distribution and missingness (structural and true) of variables used to construct the composite variable risk sexual behaviours. (DOCX)

S2 Table Sensitivity analysis comparing the primary model and the sensitivity model. (DOCX)

S3 Table Prevalence of risky sexual behaviors and related characteristics by age group among adolescent girls and young women in the 2022–2023 THIS. (DOCX)

S1 File STROBE Checklist for cross-sectional studies. (DOCX)

S1 Fig Analytical sample flow. (TIF)

S1 Data STATA do-file data analysis procedure. (DO)
