# Supplementary material for: Risky sexual behaviors and associated factors among adolescent girls and young women in the context of HIV prevention pre-exposure prophylaxis in Tanzania: Analysis of the Tanzania HIV Impact Survey 2022–2023
Source: PLOS Glob Public Health. 2026 Oct 23;6(10):e0007311. doi: 10.1371/journal.pgph.0007311 (PMC13693934; doi:10.1371/journal.pgph.0007311)
Supplement: Supplementary file 1 [file pgph.0007311.s001.docx]

**S1 Table**. Distribution and missingness (structural and true) of variables used to construct

| **Variable** | **Observed, n (%)** | **Structural missing, n (%)** | **True missing, n (%)** | **No RSB, n (%)** | **Yes**  **RSB, n (%)** | **Total, n- prior to deleting missing value on RSB** |
| --- | --- | --- | --- | --- | --- | --- |
| Age at first sexual intercourse | 3,943 (93.99) | 0 (0.00) | 252 (6.01) | 3602 (85.86) | 341(8.13) | 4,195 |
| Number of sexual partners in past 12 months | 3,686 (87.87) | 0 (0.00) | 211 (5.03) | 3722(88.72) | 262(6.25) | 4,195 |
| Condom use at last sex with a non-marital/non-cohabiting partner | 1,295 (30.87) | 2,382 (56.78) | 518*(12.35) | 2678(63.84) | 999(23.81) | 4,195 |
| **Composite RSB outcome (without condom use at last sexual intercourse)** | | | **526 (12.27)** | **2354 (56.11)** | **1315 (31.42)** | **4195** |
|  |  |  | **Excluded missing** | **2354 (56.11)** | **1315 (31.42)** | **3669** |

risky sexual behavior composite outcome.
