# Supplementary material for: Risky sexual behaviors and associated factors among adolescent girls and young women in the context of HIV prevention pre-exposure prophylaxis in Tanzania: Analysis of the Tanzania HIV Impact Survey 2022–2023
Source: PLOS Glob Public Health. 2026 Oct 23;6(10):e0007311. doi: 10.1371/journal.pgph.0007311 (PMC13693934; doi:10.1371/journal.pgph.0007311)
Supplement: Supplementary file 2 [file pgph.0007311.s002.docx]

**S2 Table: Sensitivity analysis comparing the primary model and the sensitivity model.**

**Purpose.** The primary model used three indicators to construct the composite RSB outcome. The sensitivity analysis added early first marriage/cohabitation as a fourth indicator after distinguishing structural non-applicability from true missingness and excluding records with true missing outcome data.

| **RSB indicator** | **Risk classification** | **Primary 3-component model** | **Sensitivity 4-component model** |
| --- | --- | --- | --- |
| Age at first sexual intercourse | First sex before age 15 years | Included | Included |
| Age at first marriage/cohabitation | First lived with a marital/cohabiting partner before age 15 years | Not included | Included |
| Condomless sex with a non-marital/non-cohabiting partner | Yes | Included | Included |
| Number of sexual partners in the previous 12 months | Two or more partners | Included | Included |

*For either definition, participants were classified as having RSB when at least one included indicator was present.*

**Direct comparison of the two adjusted models**

| **Age group** | **Primary model** | **Sensitivity model** | **Comparison** |
| --- | --- | --- | --- |
| **15-17 years** | n = 299; F (14,24) = 3.55 | n = 299; F (14,151) = 3.80 | AOR point estimates unchanged; CIs and p-values differed; significant factors unchanged. |
| **18-24 years** | n = 2,766; F (14,251) = 36.39 | n = 2,782; F (14,252) = 36.33 | AORs were very similar; direction and significance pattern unchanged. |

**Sensitivity analysis key findings.** No predictor changed statistical significance status. In both definitions, not being in a union and alcohol use were associated with higher odds of RSB among participants aged 15-17 years. Among those aged 18-24 years, the same eight factors remained significant: secondary+ education, parent/guardian discussion and prior HIV testing were associated with lower odds, whereas not being in a union, a health-facility visit, family-planning use, alcohol use and PrEP willingness were associated with higher odds. The primary and sensitivity outputs do not use identical survey degrees of freedom, particularly for ages 15-17 years (F denominator df 24 vs 151), and the adjusted sample for ages 18-24 increased from 2,766 to 2,782. This explains why confidence intervals and p-values can differ even when AOR point estimates are unchanged.

**S4 Table. Adjusted primary and sensitivity models for risky sexual behavior, stratified by age group**

| **Variable and category** | **Ages 15-17** **Sensitivity 4-component model** | **Used in the manuscript**  **Ages 15-17** **Primary 3-component model** | **Ages 18-24** **Sensitivity 4component model** | **Used in the manuscript**  **Ages 18-24** **Primary 3-component model** |
| --- | --- | --- | --- | --- |
| Education: Primary | 1.23 (0.45-3.38) p = 0.684 | 1.23 (0.53-2.86) p = 0.633 | 0.80 (0.56-1.14) p = 0.216 | 0.81 (0.57-1.16) p = 0.249 |
| Education: Secondary+ | 0.94 (0.29-2.99) p = 0.912 | 0.94 (0.34-2.59) p = 0.902 | **0.55 (0.37-0.81)** **p = 0.003*** | **0.56 (0.38-0.83)** **p = 0.004*** |
| Worked in previous 12 months: Yes | 0.73 (0.25-2.13) p = 0.552 | 0.73 (0.34-1.53) p = 0.399 | 0.80 (0.63-1.02) p = 0.076 | 0.81 (0.64-1.02) p = 0.076 |
| Wealth index: Middle | 0.47 (0.12-1.93) p = 0.289 | 0.47 (0.20-1.11) p = 0.085 | 0.95 (0.69-1.29) p = 0.724 | 0.96 (0.71-1.31) p = 0.814 |
| Wealth index: Rich | 0.69 (0.15-3.23) p = 0.625 | 0.69 (0.23-2.06) p = 0.499 | 0.90 (0.64-1.26) p = 0.546 | 0.91 (0.65-1.27) p = 0.571 |
| Residential location: Rural | 0.87 (0.20-3.83) p = 0.850 | 0.87 (0.35-2.17) p = 0.764 | 0.85 (0.66-1.10) p = 0.211 | 0.85 (0.66-1.10) p = 0.211 |
| **Union status: Not in union** | **8.72 (3.48-21.85)** **p < 0.001*** | **8.72 (4.33-17.55)** **p < 0.001*** | **15.13 (11.38-20.12)** **p < 0.001*** | **14.77 (11.14-19.59)** **p < 0.001*** |
| Visited a health facility: Yes | 1.29 (0.54-3.12) p = 0.557 | 1.29 (0.67-2.50) p = 0.444 | **1.30 (1.04-1.61)** **p = 0.021*** | **1.30 (1.04-1.62)** **p = 0.021*** |
| Discussed sex/HIV with parents or guardians: Yes | 0.70 (0.16-3.14) p = 0.638 | 0.70 (0.27-1.87) p = 0.480 | **0.60 (0.45-0.79)** **p < 0.001*** | **0.59 (0.44-0.78)** **p < 0.001*** |
| Currently using family planning: Yes | 1.68 (0.56-4.99) p = 0.342 | 1.68 (0.71-3.97) p = 0.237 | **1.31 (1.02-1.69)** **p = 0.033*** | **1.33 (1.04-1.69)** **p = 0.023*** |
| **Alcohol use: Yes** | **13.38 (3.11-57.49)** **p = 0.001*** | **13.38 (1.53-116.64)** **p = 0.019*** | **2.46 (1.56-3.88)** **p < 0.001*** | **2.43 (1.54-3.82)** **p < 0.001*** |
| Ever tested for HIV: Yes | 1.52 (0.68-3.42) p = 0.301 | 1.52 (0.80-2.89) p = 0.198 | **0.60 (0.43-0.84)** **p = 0.003*** | **0.62 (0.45-0.86)** **p = 0.005*** |
| PrEP awareness: Yes | 0.54 (0.13-2.19) p = 0.382 | 0.54 (0.18-1.63) p = 0.275 | 0.95 (0.59-1.54) p = 0.838 | 0.93 (0.57-1.49) p = 0.749 |
| PrEP willingness: Yes | 1.01 (0.45-2.26) p = 0.979 | 1.01 (0.57-1.78) p = 0.970 | **1.36 (1.04-1.77)** **p = 0.024*** | **1.35 (1.04-1.77)** **p = 0.025*** |

Values are adjusted odds ratios (AORs) with 95% confidence intervals; p-values are shown on the second line.
