# Supplementary material for: Risky sexual behaviors and associated factors among adolescent girls and young women in the context of HIV prevention pre-exposure prophylaxis in Tanzania: Analysis of the Tanzania HIV Impact Survey 2022–2023
Source: PLOS Glob Public Health. 2026 Oct 23;6(10):e0007311. doi: 10.1371/journal.pgph.0007311 (PMC13693934; doi:10.1371/journal.pgph.0007311)
Supplement: Supplementary file 3 [file pgph.0007311.s003.docx]

**S2 Table.** Prevalence of risky sexual behaviors and related characteristics by age group among adolescent girls and young women in the 2022-2023 THIS.

| **Sexual behaviour characteristic** | **Overall** | | **Age 15-17 years** | | **Age 18-24 years** | |
| --- | --- | --- | --- | --- | --- | --- |
|  | **n** | **Weighted % (95% CI)** | **n** | **Weighted % (95% CI)** | **n** | **Weighted % (95% CI)** |
| **Final composite risky sexual behaviour** |  |  |  |  |  |  |
| No RSB | 2,354 | 61.71 (59.70-63.68) | 141 | 38.43 (33.38-43.74) | 2,213 | 64.37 (62.36-66.33) |
| Yes RSB | 1,315 | 38.29 (36.32-40.30) | 211 | 61.57 (56.27-66.62) | 1,104 | 35.63 (33.67-37.64) |
| **Age at first sexual intercourse** |  |  |  |  |  |  |
| 0-14 years | 341 | 9.30 (8.17-10.57) | 93 | 25.80 (21.37-30.80) | 248 | 7.41 (6.43-8.53) |
| 15-17 years | 1,768 | 48.36 (46.25-50.47) | 258 | 74.20 (69.20-78.63) | 1,510 | 45.39 (43.18-47.62) |
| 18-24 years | 1,540 | 42.34 (39.81-44.91) | 0 | 0.00 (NE) | 1,540 | 47.19 (44.67-49.73) |
| **Condomless-sex measure: observation status** |  |  |  |  |  |  |
| Structurally missing | 2,337 | 61.34 (58.81-63.81) | 136 | 38.09 (31.92-44.67) | 2,201 | 63.83 (61.30-66.29) |
| Observed | 1,290 | 38.66 (36.19-41.19) | 190 | 61.91 (55.33-68.08) | 1,100 | 36.17 (33.71-38.70) |
| **Number of sexual partners in the previous 12 months** |  |  |  |  |  |  |
| 0 partners | 33 | 0.86 (0.58-1.29) | 24 | 6.06 (3.79-9.57) | 9 | 0.27 (0.13-0.55) |
| 1 partner | 3,370 | 91.80 (90.65-92.83) | 305 | 86.52 (81.58-90.30) | 3,065 | 92.41 (91.29-93.39) |
| 2 or more partners | 262 | 7.34 (6.42-8.37) | 23 | 7.41 (4.75-11.39) | 239 | 7.33 (6.39-8.39) |
| **Notes:** n= number of respondents; percentages and 95% confidence intervals are survey-weighted and account for the complex survey design. Denominators vary because missing observations were excluded. NE = confidence interval not estimable because the category contained no observations. RSB = risky sexual behavior. | | | | | | |
