# Supplementary material for: Risky sexual behaviors and associated factors among adolescent girls and young women in the context of HIV prevention pre-exposure prophylaxis in Tanzania: Analysis of the Tanzania HIV Impact Survey 2022–2023
Source: PLOS Glob Public Health. 2026 Oct 23;6(10):e0007311. doi: 10.1371/journal.pgph.0007311 (PMC13693934; doi:10.1371/journal.pgph.0007311)
Supplement: Supplementary file 5 [file pgph.0007311.s007.docx]

**Reviewer report**

*Confidential to the editor and authors*

| **Manuscript number** | PGPH-D-26-02863 |
| --- | --- |
| **Title** | Risky Sexual Behaviours and Associated Factors among Adolescent Girls and Young Women in the Context of HIV Prevention Pre-exposure Prophylaxis in Tanzania: Analysis of the Tanzania HIV Impact Survey 2022-2023 |
| **Journal** | PLOS Global Public Health |
| **Article type** | Research Article |
| **Recommendation** | Major revision |
| **Date of review** | 12 August 2026 |

# **Summary of the submission**

The authors analyse the 2022-2023 Tanzania HIV Impact Survey to estimate the prevalence of a composite risky sexual behaviour (RSB) measure among 3,482 sexually active, HIV-negative adolescent girls and young women aged 15-24, and to identify factors associated with it. They stratify by age (15-17 and 18-24), run survey-weighted logistic regression, and report that just over half the sample met the composite definition, with not being in a union emerging as the dominant correlate in both age bands.

The question matters, the data source is the right one, and a nationally representative estimate for this population is genuinely worth having. Tanzania has good survey infrastructure and comparatively few papers that use it to look at adolescent girls specifically. I would like to see this published. But I cannot recommend acceptance in its current form, for reasons that are mostly fixable and one that is not fixable without reanalysis.

# **General assessment**

My central concern is structural. The outcome variable is a composite of four indicators, two of which are defined in terms of marital or cohabiting status: marriage before age 15, and condomless sex with a non-marital or non-cohabiting partner. Union status is then entered into the regression as the principal exposure, where it returns an adjusted odds ratio of 16.92 among 18- 24-year-olds. That is not a behavioural effect; it is an artefact of an outcome that partly encodes the exposure. Any reader who works with survey data will see this immediately, and it undermines the finding the paper leads with in its abstract and conclusion.

The second problem is that the abstract does not describe the tables. Prevalence is 53.3% in the abstract and 54.1% in the Results. The age distribution is 86.9% in the abstract and 89.91% in Table 1. The alcohol finding is reported in the abstract as the opposite of what Table 2 shows. Separately, the Overall column of Table 1 sums to 3,947 rather than the stated 3,482, and PrEP use appears as 35 respondents overall but only 13 across the two age columns. These are the kinds of discrepancies that make an editor doubt the whole analysis rather than the abstract alone, so they need clearing up before anything else.

Third, the survey analysis is not described in a way anyone could reproduce. The paper says the two datasets were separately weighted and then merged, which is not how complex-survey estimation works. It does not name the weight variable, the strata or PSU variables, or the replicate weight set, and the differing denominator degrees of freedom in the Table 2 footnote suggest the age groups were analysed by dropping observations rather than with subpop(). For a secondary analysis of a public dataset, the methods section carries most of the paper’s credibility, and this one currently does not.

Beyond that, the PrEP framing is doing more work in the title and introduction than the data can support. PrEP use in this sample is 1.0% and awareness is 5.9%. A population that has essentially not encountered PrEP cannot tell us anything about risk compensation. The null PrEP findings are the expected result, not a surprising one, and the paper should say so rather than presenting them as a test of a hypothesis.

The writing itself needs a careful pass. There are fragments, incorrect connectors, at least one incorrect word with factual meaning, and several numbers in the Discussion that I could not trace to any table. I have listed the ones I found, but the authors should assume the list is not exhaustive.

# **Major points**

*These require substantive revision or reanalysis before the paper can be reconsidered.*

**M1.** Circularity between the outcome and union status. Two of the four RSB components can only be endorsed by respondents who are not married or cohabiting. Union status is then the main exposure. This is almost certainly the source of the implausibly large odds ratios (7.34 and 16.92, the latter with a lower bound of 11.41). I would ask the authors to rebuild the composite so that it does not embed union status, rerun both models, and report how much the estimate moves. If the association survives on a clean outcome, the paper is much stronger for it. If it collapses, that is itself worth reporting. Presenting union status as a stratifying variable instead would be an acceptable second-best, but only with the causal language removed throughout, including from the abstract conclusion and the recommendations.

**M2.** The authors need to reconcile the abstract with the tables. Prevalence (53.3% vs 54.1%), the proportion aged 18-24 (86.9% vs 89.91%), and the direction of the alcohol association all disagree between the abstract and the results. Rebuild the abstract from the final output. State every estimate with its referent and its confidence interval, and say explicitly whether percentages are weighted.

**M3.** Table 1 does not add up. Every variable I checked sums to 3,947 in the Overall column against a stated N of 3,482 (education 396 + 2,110 + 1,441; wealth 1,723 + 809 + 1,414; working status 1,339 + 2,608; PrEP use 3,912 + 35). The two age columns do sum correctly to 3,482. PrEP use is also irreconcilable: 35 users overall against 2 and 11 in the age strata. Please check the table against your output and explain in the footnote which columns are weighted and which are counts.

**M4.** Describe the survey analysis so it can be reproduced. Name the weight variable and justify the choice, since THIS provides distinct interview and blood-test weights and the analysis conditions on HIV test result. Give the strata and PSU variables and the jackknife replicate weight set. Confirm whether subpop() was used for the age-stratified models; if the sample was restricted with an if condition instead, the standard errors and design degrees of freedom are wrong, and the models need rerunning. Depositing the Stata do-file as supporting information would resolve this in one step.

**M5.** Resolve the condom use contradiction. The Methods state that condom use was excluded because the survey did not measure it. Condom use at last sex is nonetheless a covariate in Table 1, a component of the outcome, and a topic in the Discussion. THIS 2022-2023 does collect it. Correct the sentence and remove or clearly flag the Table 1 bivariate association, which is tautological given that the outcome was built from the same variable. The VIF of 13.89-29.23 you report is the symptom of exactly this.

**M6.** Report the missing data. The sentence explaining that respondents with missing values on outcome components were left missing is garbled and, more importantly, unquantified. Give the number missing on each component, the number dropped from the composite, and whether the analysis is complete-case. Where the missing fraction is material, add a sensitivity analysis.

**M7.** Address sparse-data instability. Alcohol use among 15- 17-year-olds gives an AOR of 0.08 with an interval of 0.01 to 0.75, in a stratum of 322. PrEP use rests on 2 respondents in the younger group and 11 in the older group, yet is carried into the multivariable model with intervals up to 24.37. Report the cell counts, use Firth penalised regression or exact methods where appropriate, and drop PrEP use from the regression, reporting it descriptively instead.

**M8.** Make the eligibility restriction visible and assess it. Requiring valid responses to all three PrEP items is a selection step and does not appear in Figure 1. Add it with the number excluded, and compare included and excluded respondents on age, education, residence and union status. The generalisability claim in the Limitations should then be softened to match.

**M9.** Correct Figure 1 and its starting denominator. The figure begins at 33,366 while the text says 33,663; only the latter makes the first subtraction work (33,663 minus 23,759 = 9,904). Note also that THIS recorded 35,960 completed individual interviews and 33,663 completed biomarker tests, so state which denominator you are using and why.

**M10.** Reconsider treating marriage before 15 as a risky sexual behaviour of the girl. Marriage at that age is child marriage, and classifying it as her behaviour attributes to her a decision she did not make. This sits badly beside the paper’s own account of structural drivers, and it contributes to the circularity in M1. At minimum, the authors should defend the choice explicitly; my preference would be to drop the component and treat child marriage as an exposure or a context variable.

**M11.** Recalibrate the PrEP framing. With 1.0% use and 5.9% awareness, this sample cannot test behavioural risk compensation. Say that directly rather than describing the null findings as surprising. The title should also come down accordingly, since it currently promises an analysis situated in the context of PrEP provision that the data cannot deliver.

**M12.** Model diagnostics. The goodness-of-fit test is misnamed (Hosmer-Lem shows) and, more to the point, no result is reported anywhere. After svy estimation, Stata’s estat gof returns the Archer-Lemeshow F-adjusted mean residual test; name it correctly and report it for each model, along with the analytic N. If the paper wants to claim that associations differ by age group, a formal interaction test in the pooled model is needed rather than a comparison of two sets of p-values.

**M13.** Reporting and declarations. PLOS treats a missing STROBE checklist for an observational study as a revise-before-review issue; complete it with line numbers and cite it in the Methods. Use the journal’s competing interests wording ("The authors have declared that no competing interests exist"), and state in the data availability statement that access is open to any researcher on the same terms and that the authors held no special privileges. Give the exact dataset name and version.

# **Minor points**

**m1.** Spelling variants drift between British and American forms across the submission, including between the Editorial Manager record and the manuscript file. Choose one and apply it everywhere.

**m2.** The abbreviation flips between RSB and SRB in at least four places, including the Discussion. The acronym list defines RSB as "Risk Sexual behaviour" and lists TDHS, which never appears. Consider deleting the acronym list and defining terms at first use, as PLOS does not usually carry one.

**m3.** PrEP is written PREP in the abstract.

**m4.** Affiliations do not resolve: four are listed, but the second author carries superscript 5, and affiliation 4 is unassigned.

**m5.** "AGYW are the cornerstone of new HIV infections" is the wrong wording. You mean they account for a disproportionate share.

**m6.** The sentence beginning "While Tanzania has made notable progress..." has no main clause. Join it to the sentence that follows.

**m7.** "The new HIV incidence among AGYW remains high, up to 80%" cannot be read as written. Name the indicator, the numerator, the denominator and the year, or delete. The same applies to "a rise from 14% in 2015/16 to 30% in 2022/23".

**m8.** Describing the difference between 1.3% at 15-19 and 4.4% at 20-24 as a rise conflates an age gradient with a time trend. Reword. Also check that 4.4% has not been carried over from the national adult prevalence figure.

**m9.** The "2026 HIV global update" needs its year checked. The most recent UNAIDS products online are the 2025 Global AIDS Update and the 2025 World AIDS Day report.

**m10.** I could not verify the claim that global PrEP uptake fell 38% between 2024 and 2025 across 62 countries. The 2025 World AIDS Day report gives country-level declines (roughly 31% in Uganda, 21% in Viet Nam, 23% in Ukraine, 64% in Burundi) rather than a single pooled figure. Give the exact source and reporting period, or replace it. Note also that your data predate these disruptions, so this belongs in context rather than in findings.

**m11.** "Whereby, studies conducted in Ethiopia..." uses a connector that does not work between sentences. Several similar slips follow ("which is like prior studies", "The latter finding", "Additionally, the differences in findings may reflect").

**m12.** The claim that PHIA surveys are conducted every five years is not accurate. THIS 2022-2023 is the second PHIA round in Tanzania and the fifth household HIV survey since 2003.

**m13.** "(Insert Table 1 here)" is an editing artefact left in the submitted file, and it names the wrong table.

**m14.** Table 2 footnote: "F(15,216) = 17:17" and "F(15,139) =2.67:" contain punctuation errors, and the footnote does not say what the F-statistic tests.

**m15.** "Residencial" appears in Figure 2 and in Table 2.

**m16.** The Discussion reports 89.0% and 85.8% for condom non-use; neither figure appears in the Results, where the corresponding values are 82.4% and 92.4%. Every number in the Discussion should trace back to a table.

**m17.** The suggestion that alcohol policy restricting sales to under-18s explains the inverse alcohol association is offered without a citation or any evidence on timing. Present it as one possibility among several, and give the measurement explanation at least equal weight.

**m18.** I would drop the word "protective" for the alcohol finding. An inverse association in cross-sectional self-report, running against the regional literature, points to differential under-reporting and a crude any-versus-none exposure measure rather than protection.

**m19.** The passage on reconceptualising RSB in the era of PrEP and U=U is the best writing in the paper and deserves a signposted subsection. It should also connect back to your own measure, and the Limitations should say plainly that the central variable inherits the limitation rather than that "a more suitable definition could not be identified".

**m20.** The conclusion is missing a conjunction ("HIV testing status alcohol use") and recommends actions that do not follow from what was tested. Tie each recommendation to a specific result.

**m21.** Reference list needs a full Vancouver pass. "Located at: CABI Databases" and similar database artefacts have been carried over from a reference manager, PMID and PMCID appear inconsistently, and the numbering in the submitted file does not run cleanly.

**m22.** Figure 3 needs axis labels, denominators and confidence intervals shown or stated in the caption. Table titles should each specify weighting and the analytic N.

**m23.** State whether a local ethics committee reviewed the secondary analysis or formally waived it, in addition to the approvals held by the parent survey.

# **Section-by-section notes**

## **Title and abstract**

Covered in M2 and M11. One additional thought: a short title that names the survey and the age band would do better in search than the current one. The abstract conclusion should also stop short of recommending age-specific strategies until the union-status finding has been shown to survive M1.

## **Introduction**

The literature is broadly the right literature and the regional comparisons are useful. The problem is that the numbers are loosely handled. Several statistics appear without an indicator, a denominator or a year; one age gradient is described as a temporal rise, and one figure (80%) cannot be read at all. The gap statement is also weaker than it needs to be: the argument that previous work looked at single behaviours or selected subgroups is a good one, but it is buried in a long sentence carrying eight citations. Give it its own two sentences.

## **Methods**

The study design paragraph describes PHIA in general terms and then leaves the specifics unstated. What the reader needs is the survey round, the fieldwork dates, the sampling frame, the response rates, the weight used and why, and the design variables. The variable definitions section is clearer, but it contains the condom use contradiction (M5), does not quantify missing data (M6), and does not define the geographical zones variable, which is derived rather than published in the survey report. The statistical analysis paragraph needs rewriting from scratch along the lines of M4 and M12.

## **Results**

The narrative reports significance but rarely reports the estimate the reader wants alongside it, and it does not distinguish weighted from unweighted quantities. The prevalence paragraph is well structured, and the confidence intervals are welcome; carry that discipline into the rest of the section. Table 1 needs the arithmetic fixed (M3), and the condom use row reconsidered (M5). Table 2 needs analytic N per model, cell counts where estimates are sparse, and the PrEP use rows removed (M7). The sentence stating that HIV testing was not associated with RSB is true only for the older group.

## **Discussion**

The comparative paragraphs work well, and the regional benchmarking is one of the paper’s strengths. Three things need attention. Numbers appear that are not in the Results (m16). Explanations are offered with more confidence than the design supports, particularly for alcohol and for the HIV testing association among 15-17 year olds, where the reassurance-after-negative-test story is plausible but untested and should be framed as a hypothesis. And the PrEP paragraph asks the data to answer a question they cannot answer (M11). The final section on reconceptualising risk is the part I would keep and expand.

## **Limitations**

Candid as far as it goes, but incomplete. Add the eligibility restriction and its consequences, the structural overlap between the exposure and the outcome, missing data, sparse-data instability in the younger stratum, and the fact that the data predate the 2025 prevention funding disruptions. The strength claim about being the first nationally representative Tanzanian study of RSB in the context of PrEP availability is hard to sustain at 1% PrEP use.

## **Declarations and reference list**

See M13 and m21. The funding statement and the acknowledgement of SPHeRE and HRB training should be checked for consistency, since two funders currently appear in different places without explanation of their respective roles.

# **Language and readability**

The manuscript reads as though several drafting passes have been layered on top of each other without a final read-through. Sentences run long and flatten out to a uniform length, connectors are used where the logic does not need them, and abstractions stand in for the specific claim. That style also happens to be what institutional screening tools now flag, so tightening it serves two purposes at once.

Three habits are worth breaking. First, participial clauses tacked onto findings to manufacture significance: "highlighting the need for", "underscoring the importance of", "reflecting broader trends". State the result and let it stand. Second, the stacked phrase of three, which appears repeatedly in the introduction and discussion and dilutes rather than strengthens. Third, hedged verbs where a plain one exists: "may potentially influence" instead of "influences", "utilise" instead of "use". Vary sentence length deliberately. A short sentence after a long one does more for readability than any amount of restructuring.

Some specific rewrites, offered as illustrations rather than as required edits:

| **As submitted** | **Suggested** |
| --- | --- |
| *AGYW are the cornerstone of new HIV infections.* | AGYW account for the largest share of new infections in Tanzania, at X% of the national total in 2022-2023. |
| *These findings highlight the need for age-specific HIV prevention strategies that integrate behavioural interventions with biomedical approaches.* | Prevention services should be differentiated by age. The 15-17 group in this sample carried the higher burden and reached PrEP least often. |
| *Therefore, this study utilizes nationally representative data from the 2022-2023 Tanzania HIV Impact Survey (THIS) to explore the prevalence and factors associated with RSB.* | We used THIS 2022-2023 to estimate national prevalence of RSB among sexually active AGYW and to test which characteristics were associated with it. |
| *The latter findings among AGYW who were not in a union may reflect the greater sexual autonomy and relationship transitions experienced during adolescence and young adulthood, a period often characterized by exploration and experimentation with sexual relationships.* | Unmarried AGYW may simply have more non-cohabiting partners, which is what two of our four outcome components measure. Until the composite is rebuilt, we cannot separate autonomy from definition. |
| *This finding underscores the need to strengthen HIV testing services by integrating post-test risk-reduction counselling, PrEP education, condom promotion, and adolescent-friendly SRH services.* | A negative test is currently an endpoint. It could be an entry point: post-test counselling, PrEP information and condom supply offered in the same visit. |

# **What would change my recommendation?**

M1 is the point on which the paper turns. If the composite outcome is rebuilt without the union-dependent components and the analysis rerun, and if M2 to M4 are dealt with so that the tables, the abstract and the described methods agree with one another, this becomes a solid contribution to a thin evidence base and I would expect to recommend acceptance. The remaining points are ordinary revision work. I am happy to look at a revised version.
