# Supplementary material for: Risky sexual behaviors and associated factors among adolescent girls and young women in the context of HIV prevention pre-exposure prophylaxis in Tanzania: Analysis of the Tanzania HIV Impact Survey 2022–2023
Source: PLOS Glob Public Health. 2026 Oct 23;6(10):e0007311. doi: 10.1371/journal.pgph.0007311 (PMC13693934; doi:10.1371/journal.pgph.0007311)
Supplement: Supplementary file 6 [file pgph.0007311.s008.docx]

PLOS Global Public Health

**Risky Sexual Behaviours and Associated Factors among Adolescent Girls and Young Women in the Context of HIV Prevention Pre-exposure Prophylaxis in Tanzania: Analysis of the Tanzania HIV Impact Survey 2022-2023**

--Manuscript Draft--

| **Manuscript Number:** | PGPH-D-26-02863 |
| --- | --- |
| **Article Type:** | Research Article |
| **Full Title:** | Risky Sexual Behaviours and Associated Factors among Adolescent Girls and Young Women in the Context of HIV Prevention Pre-exposure Prophylaxis in Tanzania: Analysis of the Tanzania HIV Impact Survey 2022-2023 |
| **Short Title:** | Risky Sexual Behaviours among Adolescent Girls and Young Women in Tanzania |
| **Corresponding Author:** | Magreth Thadei Mwakilasa, BA, MSc, MSc  University College Dublin School of Nursing Midwifery and Health Systems Dar es Salaam, TANZANIA, UNITED REPUBLIC OF |
| **Order of Authors:** | Magreth Thadei Mwakilasa, BA, MSc, MSc |
|  | Deodatus Kakoko |
|  | John Gilmore |
|  | Brynne Gilmore |
| **Keywords:** | Adolescent girls and young women; risky sexual behaviour; HIV prevention; Tanzania HIV Impact Survey; Tanzania. |
| **Abstract:** | Objective: Risky sexual behaviours remain a major contributor to HIV acquisition among adolescent girls and young women (AGYW) in Sub-Saharan Africa (SSA), particularly in Tanzania, where AGYW continue to bear a disproportionate burden of new HIV infections. Nationally representative evidence on the prevalence of risky sexual behaviour (RSB) and its associated factors among Tanzanian AGYW remains limited, particularly in the context of expanding pre-exposure prophylaxis (PrEP) choices and provision. This study examines the prevalence of risky sexual behaviour and the factors associated with it among AGYW aged 15-24 years in Tanzania, in the era of PREP provision.  Methods: A secondary analysis of nationally representative data from the 2022-2023 Tanzania HIV Impact Survey (THIS) was conducted among 3482 sexually active AGYW aged 15-24 who reported PrEP-related outcomes. Descriptive statistics summarised respondents’ characteristics and the prevalence of risky sexual behaviour. Associations between participant characteristics and risky sexual behaviour were assessed using the chi-square test, and survey-weighted multivariable logistic regression identified factors that were independently associated with risky sexual behaviour. Adjusted odds ratios (AORs) with 95% confidence intervals (CIs) were reported, and statistical significance was set at p < 0.05.  Results: Overall, 53.3% of adolescent girls and young women engaged in risky sexual behaviour; most participants were aged 18-24 years (86.9%) and had primary education (52.1%). Across both age groups, not being in a marital/union relationship was associated with increased odds of risky sexual behaviour among adolescents aged 15-17 years (AOR = 7.34, 95% CI: 3.58-15.04; p < 0.001) and young women  aged 18-24 years (AOR = 16.92, 95% CI: 11.41-25.09; p < 0.001). Among adolescent girls aged 15-17 years, ever testing for HIV was also associated with increased odds (AOR = 2.00, 95% CI: 1.03-3.91; p = 0.041), whereas alcohol non-use was associated with lower odds of risky sexual behaviour in both age groups (15-17 years: AOR = 0.08, 95% CI: 0.01-0.75; p = 0.027; 18-24 years: AOR = 0.46, 95% CI: 0.29-0.73; p =  0.001). All other variables were not statistically significant after adjustment.Conclusion: Risky sexual behaviour was common among adolescent girls and young women in Tanzania and varied by age group. These findings highlight the need for age-specific HIV prevention strategies that integrate behavioural interventions with biomedical approaches, particularly for adolescent girls and young women who are not in unions. |

*Powered by Editorial Manager® and ProduXion Manager® from Aries Systems Corporation*

| **Additional Information:** |  |
| --- | --- |
| **Question** | **Response** |
| **Financial Disclosure**  Enter a financial disclosure statement that describes the sources of funding for the work included in this submission and the role the funder(s) played. This includes grants and any commercial funding of the work or authors.  This statement will be typeset if the manuscript is accepted for publication.  *Please review the* [*submission guidelines*](http://journals.plos.org/globalpublichealth/s/submission-guidelines#loc-financial-disclosure-statement) *and the instructions link below for detailed requirements and guidance.* | The Ministry of Education, Science and Technology (MoEST) funded this study through the Higher Education for Economic Transformation (HEET) project (Grant No.  P166415). The grant was awarded to Muhimbili University of Health and Allied Sciences (MUHAS), which served as the implementing institution. The content is solely the responsibility of the authors and does not necessarily reflect the official views of the funder. The funder had no role in the study design, data collection and analysis, decision to publish, or preparation of the manuscript. |
| **Competing Interests**  On behalf of all authors, disclose any competing interests that could be perceived to bias this work.  This statement will be typeset if the manuscript is accepted for publication.  *Please review the instructions link below and PLOS Global Public Health’s* [*competing interests*](https://journals.plos.org/globalpublichealth/s/competing-interests) *policy to determine what information must be disclosed at submission.* | The author(s) declared no known financial conflicts of interest with respect to the research, authorship, and/or publication that could have influenced the work reported in this paper. |
| **Data Availability**  Before publication, Authors are required to make fully available and without restriction all data underlying their findings. Sharing the data underlying your findings increases the visibility, credibility, and impact of your work, while supporting transparency and future scientific discovery. Please see our [PLOS Data](https://journals.plos.org/globalpublichealth/s/data-availability) [Policy page](https://journals.plos.org/globalpublichealth/s/data-availability) for detailed information on | Data from the Tanzania HIV Impact Survey (THIS/PHIA) are publicly available, and researchers may request access by submitting a brief data analysis plan and study objectives through the PHIA data portal: https://phia-data.icap.columbia.edu/ |

*Powered by Editorial Manager® and ProduXion Manager® from Aries Systems Corporation*

| this policy.  A **Data Availability Statement**, detailing where the data can be accessed, is required at first submission. Insert your Data Availability Statement in the box below.  Please see the [data reporting](https://journals.plos.org/globalpublichealth/s/submission-guidelines#loc-style-and-format) section of our submission guidelines for instructions on what you need to include in your Data Availability Statement. Examples of acceptable Data Availability Statements are included here for reference:   1. The minimal data set [and accompanying code, where generated] is available at [repository name] via [DOI hyperlink] 2. No data was generated by this study. The following existing data sources were used: [dataset name] from [dataset location] available via [web address or DOI hyperlink]. 3. For Study Protocols: No datasets were generated or analysed during the current study. All relevant data from this study will be made available upon study completion.   *PLOS allows rare exemptions to address legal and ethical concerns. If you have legal or ethical restrictions, please use the box below to detail these in full sentences for the Journal team to consider.*  This statement will be typeset if the manuscript is accepted for publication. |  |
| --- | --- |

*Powered by Editorial Manager® and ProduXion Manager® from Aries Systems Corporation*

Manuscript	[Click here to access/download;Manuscript;20260723Plos](https://www2.cloud.editorialmanager.com/pgph/download.aspx?id=851684&guid=5e237213-b3ed-44bf-a97e-03964aff84f5&scheme=1)[<https://www2.cloud.editorialmanager.com/pgph/download.aspx?id=851684&guid=5e237213-b3ed-44bf-a97e-03964aff84f5&scheme=1> GPH Manuscript with author details.docx](https://www2.cloud.editorialmanager.com/pgph/download.aspx?id=851684&guid=5e237213-b3ed-44bf-a97e-03964aff84f5&scheme=1)

1 **Risky Sexual Behaviors and Associated Factors among Adolescent Girls and Young**
2 **Women in the Context of HIV Prevention Pre-exposure Prophylaxis in Tanzania:**
3 **Analysis of the Tanzania HIV Impact Survey 2022-2023**
4 Magreth Thadei Mwakilasa^1&2&3*^, Deodatus Kakoko^5^, John Gilmore^1&3^ Brynne Gilmore^1&3^

# 5 Corresponding author*

6 **Name: Magreth Thadei Mwakilasa**
7 **Email:** [margareth.thadei@gmail.com](mailto:margareth.thadei@gmail.com) or [magreth.mwakilasa@ucdconnect.ie](mailto:magreth.mwakilasa@ucdconnect.ie)

# 8 Authors Affiliations

9 1. University College Dublin, School of Nursing, Midwifery and Health Systems, Dublin,
10 Ireland.
11 2. Muhimbili University of Health and Allied Sciences, Dar es Salaam, United Republic
12 of Tanzania.
13 3. UCD Centre for Interdisciplinary Research, Education and Innovation in Health
14 Systems, University College Dublin.
15 4. Muhimbili University of Health and Allied Sciences, School of Public Health and
16 Social Sciences, Dar es Salaam, United Republic of Tanzania.

# 17 Abstract

18 **Objective:** Risky sexual behaviors remain a major contributor to HIV acquisition among
19 adolescent girls and young women (AGYW) in Sub-Saharan Africa (SSA), particularly in
20 Tanzania, where AGYW continue to bear a disproportionate burden of new HIV infections.
21 Nationally representative evidence on the prevalence of risky sexual behaviour (RSB) and its
22 associated factors among Tanzanian AGYW remains limited, particularly in the context of
23 expanding pre-exposure prophylaxis (PrEP) choices and provision. This study examines the
24 prevalence of risky sexual behaviour and the factors associated with it among AGYW aged 15-
25 24 years in Tanzania, in the era of PREP provision.
26 **Methods:** A secondary analysis of nationally representative data from the 2022-2023 Tanzania
27 HIV Impact Survey (THIS) was conducted among 3482 sexually active AGYW aged 15-24
28 who reported PrEP-related outcomes. Descriptive statistics	summarize respondents’
29 characteristics and the prevalence of risky sexual behaviour. Associations between participant
30 characteristics and risky sexual behaviour were assessed using the chi-square test, and survey-
31 weighted multivariable logistic regression identified factors that were independently associated
32 with risky sexual behaviour. Adjusted odds ratios (AORs) with 95% confidence intervals (CIs)
33 were reported, and statistical significance was set at p < 0.05.
34 **Results:** Overall, 53.3% of adolescent girls and young women engaged in risky sexual
35 behaviour; most participants were aged 18-24 years (86.9%) and had primary education
36 (52.1%). Across both age groups, not being in a marital/union relationship was associated with
37 increased odds of risky sexual behaviour among adolescents aged 15-17 years (AOR = 7.34,
38 95% CI: 3.58-15.04; p < 0.001) and young women aged 18-24 years (AOR = 16.92, 95% CI:
39 11.41-25.09; p < 0.001). Among adolescent girls aged 15-17 years, ever testing for HIV was
40 also associated with increased odds (AOR = 2.00, 95% CI: 1.03-3.91; p = 0.041), whereas
41 alcohol non-use was associated with lower odds of risky sexual behaviour in both age groups
42 (15-17 years: AOR = 0.08, 95% CI: 0.01-0.75; p = 0.027; 18-24 years: AOR = 0.46, 95% CI:
43 0.29-0.73; p = 0.001). All other variables were not statistically significant after adjustment.
44 **Conclusion:** Risky sexual behaviour was common among adolescent girls and young women
45 in Tanzania and varied by age group. These findings highlight the need for age-specific HIV
46 prevention strategies that integrate behavioral interventions with biomedical approaches,
47 particularly for adolescent girls and young women who are not in unions**.**
48 **Keywords**: Adolescent girls and young women; risky sexual behaviour; HIV prevention;
49 Tanzania HIV Impact Survey; Tanzania.

# 50 ACRONYMS

51 SSA	Sub-Saharan Africa
52 AGYW	Adolescent Girls and Young Women
53 PrEP	Pre- Exposure Prophylaxis
54 TDHS	Tanzania Demographic Health Survey
55 THIS	Tanzania HIV Impact Survey
56 PHIA	Population-Based HIV Impact Assessment
57 SRH	Sexual and Reproductive Health
58 RSB	Risk Sexual behaviour
59 STIs	Sexually Transmitted Infections

# 60 Introduction

61 Risky sexual behaviors (RSB) among adolescent girls and young women (AGYW) remain a
62 major global public health concern, despite decades of biomedical, behavioral, and structural
63 interventions to mitigate them (1–5). RSBs in the context of HIV prevention are defined “*Any*
64 *sexual behaviour that increases the probability of exposure to HIV, including but not limited*
65 *to unprotected genital contact (oral, anal, or vaginal), with or without penetration, orgasm, or*
66 *ejaculation; concurrent sexual partners or multiple sequential sexual partners; sexual activity*
67 *under the influence of drugs and/or alcohol; sexual activity in exchange for emotional support*
68 *or material goods (e.g., money, drugs); and/or sexual activity with a partner of unknown HIV*
69 *status” (1).* The above indicators are also used by global, international, and national bodies as
70 indicators of RSB (2–4). Sub-Saharan Africa (SSA) bears the heaviest HIV burden among
71 AGYW. The 2026 HIV global update shows that AGYW are two to three times more likely to
72 acquire HIV than their male counterparts in SSA (5). The HIV incidence in SSA mirrors what
73 is taking place in Tanzania, where AGYW are the cornerstone of new HIV infections. While
74 Tanzania has made notable progress in HIV treatment and testing coverage in achieving the
75 UNAIDS 95-95-95 targets. The new HIV incidence among AGYW remains high, up to 80%,
76 and recent data suggest a rise from 14% in 2015/16 to 30% in 2022/23, a concerning upward
77 trend (3,6,7). Furthermore, among AGYW, HIV prevalence is rising from 1.3% among
78 adolescent girls aged 15-19 years to 4.4% among young women aged 20-24 years, and AGYW
79 account for the largest share of new infections nationally (4). These incidences are occurring
80 in the context of increasing availability of biomedical HIV prevention technologies, such as
81 pre-exposure prophylaxis (PrEP), alongside expanding PrEP choices and more comprehensive,
82 integrated sexual and reproductive health (SRH) and HIV prevention services (2,8,9).
83 In SSA, AGYW are particularly vulnerable to HIV due to interacting biological, structural, and
84 behavioral factors, including gender inequality, poverty, limited educational opportunities,
85 unequal power in relationships, stigma surrounding sexual and reproductive health, and limited
86 access to youth-friendly health services and information (10–16). Together, these factors
87 increase the likelihood of risky sexual behaviors, including early sexual debut, inconsistent
88 condom use, transactional sex, and age-disparate relationships (10–16). These structural and
89 behavioral issues are major contributors to the persistently high HIV incidence among AGYW,
90 despite significant investments in prevention programmes across the region for a long time
91 (15–17). Therefore, the current study examines socio-demographic, behavioral, and PrEP-
92 related factors and their relationships with RSB.
93 Empirical evidence from SSA consistently shows that RSB among AGYW are prevalent. A
94 study carried out in Zambia shows that among sexually active female adolescents, 71.1%
95 reported at least one RSB, including early sexual debut, non-use of condoms, transactional sex,
96 alcohol use at last sex, or multiple partners (17). In Uganda, 79.6% of sexually active AGYW
97 reported inconsistent or no condom use, and 24.2% reported two or more sexual partners in the
98 previous year, with out-of-school AGYW showing riskier profiles than those in school (18).
99 Whereby, studies conducted in Ethiopia, Rwanda, and South Africa link RSB to younger age,
100 alcohol use, weak parental or social support, peer pressure, schooling disadvantage, residence,
101 and community-level conditions (19–21). Tanzania-specific evidence also shows that
102 transactional sex and age-disparate partnerships are common among AGYW, reported at 35%
103 and 28%, respectively, in a large prevention programme dataset (22,23). Furthermore, a recent
104 nationwide analysis found that 17.4% of female youth initiated sexual intercourse before age
105 18 years, with lower education strongly associated with early sexual debut (24,25). Despite the
106 global HIV prevention recommendations for comprehensive SRH services to reduce risk,
107 which include HIV testing, consistent condom use, sexuality education, and PrEP for
108 individuals at substantial risk of HIV infection, RSB persist across the SSA.
109 Despite growing evidence on SRB among AGYW, nationally representative evidence
110 describing the overall prevalence of SRB and its associated socio-factors among Tanzanian
111 AGYW aged 15-24 years in the context of PrEP availability remains limited. While the
112 introduction of PrEP for HIV prevention has reshaped the sexual landscape around the world,
113 particularly in terms of sexual behaviour, practices, and preferences (26). In the era of
114 increasing PrEP use, research has raised concerns about actual or perceived behavioral risk
115 compensation following PrEP initiation, that AGYW's perceived protection from PrEP may
116 influence engagement in RSB (27–29); however, the evidence remains limited, particularly
117 among AGYW.
118 Additionally, previously reviewed studies on RSB were not conducted in the context of PrEP
119 provision, with most focusing specifically on single behaviors or related outcomes such as early
120 sexual initiation, HIV testing, HIV risk perception, or Sexual Transmitted Infection (STIs)
121 burden or selected population subgroups or local settings such as in-school adolescents, higher-
122 learning students, pregnant adolescents, rural adolescents and programme-enrolled AGYW
123 (23,25,30–34), which limits the generalizability of their findings. Therefore, this study utilizes
124 nationally representative data from the 2022-2023 Tanzania HIV Impact Survey (THIS**)** to
125 explore the prevalence and factors associated with RSB among Tanzanian sexually active
126 AGYW aged 15-24 years in the context of PrEP provision. Understanding the latter in the
127 context of PrEP provision is essential for informing and designing equitable and evidence-
128 based behavioral support services alongside HIV prevention and SRH programmes.

# 129 Methods

130 **Study design**
131 This study utilizes secondary data from the Population-based HIV Impact Assessment (PHIA),
132 specifically the 2022/2023 THIS dataset (7). PHIA surveys are cross-sectional, nationally
133 representative; household-based studies conducted every five years to produce population-
134 level evidence on HIV. A two-stage cluster sampling design is used to examine key HIV
135 indicators and to assess the development and impact of national HIV response programmes.
136 The surveys include individuals aged 15 and above and use interviewer-administered
137 questionnaires to collect comprehensive demographic, socio-economic, behavioral, and
138 clinical data (7). Furthermore, home-based HIV testing and counselling are conducted in line
139 with national testing protocols, and laboratory confirmation and HIV viral load assessment are
140 performed for individuals who test positive for HIV. More detailed information on the THIS
141 survey design, including sampling, recruitment, and data collection procedures, has been
142 published elsewhere (7). Permission to access the dataset for this analysis was granted after
143 submission on October 17, 2024, and approval on October 24, 2024. Also, because the current
144 study relies on secondary data, the researchers had no direct contact with participants and were
145 unable to identify them in the survey.

# 146 Study population

147 To answer the research question, this study included data from participants in the PHIA-THIS
148 2022/2023 dataset who met the following criteria: were 15-24 years old, were female, tested
149 negative for HIV, reported being sexually active, and responded to all three survey questions
150 on PrEP awareness, willingness, and use. Data from respondents who did not meet all these
151 criteria were excluded. The recent survey included 33,663 respondents aged 15 years and older.
152 After applying the inclusion criteria, a sample size of 3482 sexually active AGYW was
153 obtained. See ***Figure 1*** below for more details.

154

155

# Figure 1: Participant flow diagram

156

157

# Variable of measurement

158 **Dependent variable**
159 The dependent variable was the composite RSB, treated as a binary variable. Participants who
160 reported at least one of these behaviors were classified as engaging in RSB (coded as "Yes").
161 In contrast, those who did not report any of the risk indicators were classified as not engaging
162 in RSB (coded “No”). Participants were considered engaging in risky RSB if they reported any
163 of the following as “Yes”: (1) sexual debut before the age of 15 years; (2) marriage before the
164 age of 15 years; (3) having multiple sexual partners in the previous 12 months; and (4) engaging
165 in sexual intercourse without a condom with a non-marital or non-cohabiting partner in the past
166 12 months. Those who did not experience any of the outcomes above or did not respond and
167 were missing values in each of the included variables, remained missing, and were not
168 converted to "No risk behaviour". Other important indicators of RSB, such as condom use,
169 transactional sex, sex under the influence of substances, and age-disparate relationships, were
170 excluded because the survey did not measure them.

# 171 Independent variables

172 Independent variables of interest encompassed a range of socio-demographic, behavioral, and
173 PrEP-related factors. These included age (15-17/18-24), educational attainment (no
174 education/Primary/secondary+), employment status in the past 12 months, place of residence
175 (urban, rural), union status (in a union or not in a union), wealth status (poor/middle/rich), and
176 geographical zones. Behavioral variables included health facility attendance in the previous 12
177 months, current family planning use, discussion about sex or HIV with a parent or guardian,
178 ever testing for HIV, and alcohol use, all measured as binary variables (yes/no). PrEP-related
179 indicators included PrEP awareness, willingness, and use, measured in binary (yes/no). During
180 each PHIA survey, PrEP was described as a daily medication taken to prevent HIV infection
181 before potential exposure. All respondents who reported being HIV-negative were asked
182 whether they had previously heard of PrEP (awareness). Those who were aware were asked
183 whether they had ever used PrEP, and those who had never used it, including respondents
184 introduced to PrEP during the interview, were asked about their willingness to use it for HIV
185 prevention (willingness). See ***Figure 2*** below for more details.

# 186 Figure 2: A conceptual framework of factors associated with risky sexual behaviors

187

188

# among AGYW in Tanzania.

189

190

# Statistical analysis

191 Data analysis was conducted using Stata version 18.0. Biomarker data were merged with
192 individual-level survey data and geospatial data for this analysis. Before merging, both datasets
193 were separately weighted using survey weights and jackknife variance estimation. The
194 weighted datasets were then combined into a single analytical file. Descriptive statistics were
195 computed for the weighted samples to summarize respondent characteristics and the prevalence
196 of RSB. Grouped bar graphs were used to present the prevalence of RSB. Chi-square tests were
197 subsequently performed to examine associations between independent factors and the RSB.
198 Crude odds ratios (CORs) and adjusted odds ratios (AORs), with corresponding 95%
199 confidence intervals (CIs), were estimated using logistic regression models to identify factors
200 associated with RSB. Two models were run, one for girls aged 15-17 and the second for 18-
201 24-year-olds. Statistical significance was determined using a p-value threshold of <0.05.
202 Multicollinearity among the explanatory variables was assessed using variance inflation factors
203 (VIFs). All variables had acceptable VIF values (<5), indicating no evidence of problematic
204 multicollinearity, except for condom use at last sexual intercourse (VIF range: 13.89-29.23).
205 Because condom use was one of the indicators used to construct the composite RSB outcome,
206 it was excluded from the multivariable logistic regression to avoid structural multicollinearity
207 and over-adjustment. Model goodness-of-fit was assessed using the Hosmer-Lem shows test
208 (*estat gof*).

# 209 Results

210 **Table 1** shows that most participants aged 18-24 years (86.9%), had primary education
211 (52.1%), worked in the previous 12 months (66.8%), and lived in rural areas (60.0%). Among
212 adolescent girls aged 15-17 years, RSB was significantly associated only with union status (F

213	= 36.96, *p* < 0.001) and condom use at last sexual intercourse (F = 20.38, *p* < 0.001). Among

214	young women aged 18-24 years, significant associations were observed for education level (F

215	= 40.15, *p* < 0.001), wealth index (F = 37.19, *p* < 0.001), place of residence (F = 46.08, *p* <

216 0.001), union status (F = 373.35, *p* < 0.001), condom use at last sexual intercourse (F = 14.44,
217 *p* < 0.001), and HIV testing (F = 49.78, *p* < 0.001). The remaining variables showed no
218 significant association with RSB.

# Table 1. Overall characteristics of adolescent girls and young women and bivariate association between participant characteristics and risky sexual behaviour by age group (15-17 and 18-24), Tanzania, 2022-2023.

| **Variable and categories names** | **Overall** | **Adolescent girls 15-17 years**  **n=322 (10.09%)** | | | | **18-24 years**  **n=3160 (89.91%)** | | | |
| --- | --- | --- | --- | --- | --- | --- | --- | --- | --- |
|  | **N=**3482) **n (%)** | **RSB**  **No**  **n (%)** | **RSB**  **Yes**  **n (%)** | **Adju sted**  **F** | **P-**  **value** | **RSB**  **No**  **n (%)** | **RSB**  **Yes**  **n (%)** | **Adjuste d F** | **P-value** |
| **Education level** |  |  |  |  |  |  |  |  |  |
| No education | 396 (9.4) | 18 (45.2) | 20 (54.8) | 0.56 | 0.571 | 159 (58.7) | 104 (41.4) | 40.15 | <0.001 |
| Primary | 2,110 (52.1) | 89 (41.8) | 110 (58.2) |  |  | 668 (54.0) | 506 (46.0) |  |  |
| Secondary or higher | 1,441 (38.5) | 26 (35.4) | 45 (64.6) |  |  | 202 (30.3) | 429 (69.7) |  |  |
| **Working status** |  |  |  |  |  |  |  |  |  |
| No | 1,339 (33.2) | 33 (35.9) | 47 (64.1) | 0.66 | 0.417 | 364 (45.3) | 422 (54.7) | 0.75 | 0.387 |
| Yes | 2,608 (66.8) | 100(42.3) | 128 (57.7) |  |  | 665 (47.6) | 617 (52.4) |  |  |
| **Wealth index** |  |  |  |  |  |  |  |  |  |
| Poor | 1,723 (41.3) | 80 (42.4) | 98 (57.6) | 1.42 | 0.243 | 593 (58.0) | 394 (42.0) | 37.19 | <0.001 |

| Middle | 809 (19.4) | 33 (46.1) | 33 (53.9) |  |  | 210 (47.7) | 217 (52.3) |  |  |
| --- | --- | --- | --- | --- | --- | --- | --- | --- | --- |
| Rich | 1,414 (39.3) | 20 (31.0) | 44 (69.0) |  |  | 226 (31.8) | 428 (68.2) |  |  |
| Missing | 1 (0.02) | – | – |  |  | – | – |  |  |
| **Residence location** |  |  |  |  |  |  |  |  |  |
| Urban | 1,428 (40.0) | 30 (34.9) | 53 (65.1) | 1.26 | 0.264 | 253 (33.7) | 443 (66.3) | 46.08 | <0.001 |
| Rural | 2,519 (60.0) | 103(42.9) | 122 (57.1) |  |  | 776 (54.6) | 596 (45.4) |  |  |
| **Geographical zones** |  |  |  |  |  |  |  |  |  |
| Central | 397 (9.0) | 13 (39.7) | 18 (60.3) | 1.18 | 0.312 | 128 (57.4) | 93 (42.6) | 5.67 | <0.001 |
| Northern | 200 (9.7) | 1 (9.0) | 8 (91.0) |  |  | 36 (34.1) | 65 (65.9) |  |  |
| Western | 293 (9.2) | 11 (38.7) | 18 (61.3) |  |  | 84 (54.2) | 78 (45.8) |  |  |
| Lake | 1,198 (31.1) | 56 (44.2) | 61 (55.8) |  |  | 326 (51.8) | 288 (48.2) |  |  |
| Southern Highlands | 429 (5.4) | 6 (31.7) | 17 (68.3) |  |  | 100 (47.9) | 129 (52.1) |  |  |
| Southern | 117 (3.7) | 3 (30.9) | 5 (69.1) |  |  | 21 (27.6) | 50 (72.4) |  |  |
| Eastern | 623 (19.5) | 19 (39.7) | 25 (60.3) |  |  | 145 (34.5) | 200 (65.5) |  |  |
| South-West Highlands | 550 (10.1) | 22 (53.2) | 20 (46.8) |  |  | 162 (56.4) | 120 (43.6) |  |  |
| Zanzibar | 140 (2.3) | 2 (44.5) | 3 (55.5) |  |  | 27 (56.1) | 16 (43.9) |  |  |

| **Union status** |  |  |  |  |  |  |  |  |  |
| --- | --- | --- | --- | --- | --- | --- | --- | --- | --- |
| In union | 2,588 (62.2) | 86 (64.4) | 45 (35.6) | 36.96 | <0.00  1 | 967 (66.8) | 449 (33.2) | 373.35 | <0.001 |
| Not in union | 1,347 (37.5) | 47 (24.7) | 130 (75.3) |  |  | 61 (9.3) | 590 (90.7) |  |  |
| Missing | 12 (0.3) | – | – |  |  | – | – |  |  |
| **Health facility visits**  **(12 months)** |  |  |  |  |  |  |  |  |  |
| No | 2,183 (54.5) | 62 (43.4) | 76 (56.6) | 0.91 | 0.341 | 604 (49.6) | 566 (50.4) | 7.04 | 0.009 |
| Yes | 1,764 (45.5) | 71 (38.2) | 99 (61.8) |  |  | 425 (43.1) | 473 (56.9) |  |  |
| **Discussed sex with**  **parent/guardian** |  |  |  |  |  |  |  |  |  |
| No | 3,062 (76.1) | 112 (43.2) | 132 (56.8) | 2.19 | 0.141 | 831 (47.9) | 809 (52.1) | 2.35 | 0.127 |
| Yes | 885 (23.9) | 21 (31.6) | 43 (68.4) |  |  | 198 (42.9) | 230 (57.1) |  |  |
| **Discussed HIV with**  **parent/guardian** |  |  |  |  |  |  |  |  |  |
| No | 2,851 (70.7) | 109(43.7) | 128 (56.3) | 3.03 | 0.084 | 784 (48.6) | 735 (51.4) | 5.38 | 0.021 |
| Yes | 1,096 (29.3) | 24 (31.3) | 47 (68.7) |  |  | 245 (42.0) | 304 (58.0) |  |  |

| **Family planning use** |  |  |  |  |  |  |  |  |  |
| --- | --- | --- | --- | --- | --- | --- | --- | --- | --- |
| No | 1,090 (26.4) | 17 (41.0) | 20 (59.0) | 0.23 | 0.631 | 328 (54.6) | 254 (45.4) | 16.11 | <0.001 |
| Yes | 2,281 (59.5) | 88 (36.8) | 134 (63.2) |  |  | 513 (40.7) | 653 (59.3) |  |  |
| Missing | 576 (14.2) | – | – |  |  | – | – |  |  |
| **Ever tested for HIV** |  |  |  |  |  |  |  |  |  |
| No | 665 (18.2) | 62 (42.7) | 74 (57.3) | 0.44 | 0.509 | 67 (25.1) | 169 (74.9) | 49.78 | <0.001 |
| Yes | 3,282 (81.8) | 71 (38.9) | 101 (61.1) |  |  | 962 (49.9) | 870 (50.1) |  |  |
| **Alcohol use** |  |  |  |  |  |  |  |  |  |
| No | 3,683 (93.3) | 130(41.8) | 163 (58.2) | 2.15 | 0.145 | 960 (47.8) | 928 (52.2) | 6.62 | 0.011 |
| Yes | 264 (6.7) | 3 (18.7) | 12 (81.3) |  |  | 69 (35.8) | 111 (64.2) |  |  |
| **Condom uses at last**  **sex** |  |  |  |  |  |  |  |  |  |
| Used | 361 (9.4) | 41 (77.2) | 11 (22.8) | 20.38 | <0.00  1 | 104 (70.7) | 46 (29.3) | 14.44 | <0.001 |
| Did not use | 3,099 (77.5) | 91 (32.6) | 164 (67.4) |  |  | 922 (48.3) | 989 (51.7) |  |  |
| Missing | 251 (7.0) | 1 (100.0) | 0 (0.0) |  |  | 3 (42.9) | 4 (57.1) |  |  |
| **PrEP awareness** |  |  |  |  |  |  |  |  |  |

| No | 3,720 (94.1) | 124(39.8) | 168 (60.2) | 1.23 | 0.269 | 987 (47.2) | 973 (52.8) | 2.42 | 0.121 |
| --- | --- | --- | --- | --- | --- | --- | --- | --- | --- |
| Yes | 227 (5.9) | 9 (53.9) | 7 (46.1) |  |  | 42 (38.7) | 66 (61.3) |  |  |
| **PrEP willingness** |  |  |  |  |  |  |  |  |  |
| No | 2,514 (63.1) | 86 (40.4) | 111 (59.6) | 0.00 | 0.967 | 659 (49.2) | 605 (50.8) | 4.76 | 0.030 |
| Yes | 1,433 (36.9) | 47 (42.3) | 64 (57.7) |  |  | 370 (46.0) | 434 (54.0) |  |  |
| **PrEP use** |  |  |  |  |  |  |  |  |  |
| No | 3,912 (99.0) | 132 (40.4) | 174 (59.6) | 0.44 | 0.509 | 1,027 (49.9) | 1,030 (50.1) | 2.53 | 0.113 |
| Yes | 35 (1.0) | 1 (50.0) | 1 (50.0) |  |  | 2 (18.2) | 9 (81.8) |  |  |
| ***Abbreviations:*** *RSB = Risky sexual behaviour; PrEP = pre-exposure prophylaxis. Design-adjusted F statistics and P-values are from the survey-*  *adjusted Pearson χ² test. Statistical significance was defined as* P *< 0.05, N= Number, (%) = Percentages* | | | | | | | | | |

1 **Prevalence of risky sexual behavior in Tanzania**
2 Overall, 54.1% (95% CI: 51.2-57.1) of AGYW reported engaging in RSB. The prevalence was
3 higher among adolescent girls aged 15-17 years (59.6%, 95% CI: 53.3-65.6) than among young
4 women aged 18-24 years (53.2%, 95% CI: 50.2-56.3). In both age groups, most participants
5 initiated sexual intercourse between 15-17 years (79.0%, 95% CI: 73.7-83.5 vs. 67.4%, 95%
6 CI: 64.6-70.1), did not use a condom at their last sexual intercourse (82.4%, 95% CI: 76.8-86.9
7 vs. 92.4%, 95% CI: 90.6-93.9), and reported having one sexual partner in the previous 12
8 months (91.6%, 95% CI: 87.0-94.7 vs. 89.0%, 95% CI: 87.3-90.5). Adolescent girls aged 15-
9 17 years were also more likely to report a sexual debut before age 15 years (21.0%, 95% CI:
10 16.5-26.3) than young women aged 18-24 years (10.8%, 95% CI: 9.4-12.4). Further details are
11 presented in ***Supplementary Table 1*** and ***Figure 3*** below.
12 **Figure 3. Overall and age-specific prevalence of sexual behaviour characteristics among**
13 **adolescent girls and young women.**
14

15 **Factors associated with risky sexual behaviour among AGYW in Tanzania**
16 **Table 2** below shows that among adolescent girls aged 15-17 years, not being in a union (AOR

17	= 7.34, 95% CI: 3.58-15.04, p < 0.001) and ever testing for HIV (AOR = 2.00, 95% CI: 1.03-

18 3.91, p = 0.041) increased the odds of engaging in RSB, whereas alcohol use (AOR = 0.08,
19 95% CI: 0.01-0.75, p = 0.027) was a protective factor. Among young women aged 18-24 years,
20 not being in a union (AOR = 16.92, 95% CI: 11.41-25.09, p < 0.001) increased the odds of
21 engaging in RSB, whereas alcohol use (AOR = 0.46, 95% CI: 0.29-0.73, p = 0.001) remained
22 significantly associated in the multivariate model. Education, wealth, residence, HIV testing,
23 family planning, and PrEP-related variables were not associated with RSB.
24 ***(Insert Table 1 here)***

# Table 2. Factors associated with risky sexual behaviour among AGYW aged 15-17 and 18-24 years in Tanzania: Survey-weighted logistic regression analysis (N=3,482)

|  | **Adolescent girls 15-17 years** | | | | **Young women 18-24 years** | | | |
| --- | --- | --- | --- | --- | --- | --- | --- | --- |
| **Variable and categories** | COR **(95% CI)** | ***p*-value** | **AORs (95% CI)** | ***p*-value** | COR **(95% CI)** | ***p*-value** | **AORs (95% CI)** | ***p*-value** |
| **Education** |  |  |  |  |  |  |  |  |
| No education | **Reference** |  |  |  |  |  |  |  |
| Primary | 1.15 (0.52-2.53) | 0.734 | 0.96 (0.40-2.31) | 0.930 | 1.21 (0.89-1.65) | 0.225 | 0.89 (0.58-1.36) | 0.595 |
| Secondary**+** | 1.50 (0.65-3.45) | 0.334 | 0.88 (0.32-2.44) | 0.802 | 3.26 (2.33-4.55) | <0.001***** | 0.97 (0.59-1.60) | 0.916 |
| **Worked in previous 12**  **months** |  |  |  |  |  |  |  |  |
| No | **Reference** |  |  |  |  |  |  |  |
| Yes | 0.77 (0.40-1.47) | 0.417 | 0.66 (0.32-1.40) | 0.278 | 0.91 (0.73-1.13) | 0.387 | 0.85 (0.63-1.13) | 0.262 |
| **Wealth index** |  |  |  |  |  |  |  |  |
| Poor | **Reference** |  |  |  |  |  |  |  |
| Middle | 0.86 (0.45-1.65) | 0.653 | 0.49 (0.21-1.17) | 0.107 | 1.51 (1.16-1.98) | 0.003***** | 1.05 (0.69-1.60) | 0.808 |
| Rich | 1.64 (0.81-3.32) | 0.167 | 0.68 (0.22-2.10) | 0.502 | 2.96 (2.26-3.88) | <0.001***** | 1.19 (0.76-1.85) | 0.440 |

| **Residencial location** |  |  |  |  |  |  |  |  |
| --- | --- | --- | --- | --- | --- | --- | --- | --- |
| Rural | **Reference** |  |  |  |  |  |  |  |
| Urban | 1.40 (0.77-2.53) | 0.265 | 1.08 (0.42-2.78) | 0.867 | 2.36 (1.83-3.03) | <0.001***** | 1.27 (0.91-1.79) | 0.161 |
| **Union status** |  |  |  |  |  |  |  |  |
| In union | **Reference** |  |  |  |  |  |  |  |
| Not in union | 5.52 (3.11-9.80) | <0.001***** | 7.34 (3.58-15.04) | <0.001***** | 19.67(13.75-28.16) | <0.001***** | 16.92 (11.41-25.09) | <0.001* |
| **Visited health facility** |  |  |  |  |  |  |  |  |
| No | **Reference** |  |  |  |  |  |  |  |
| Yes | 1.24 (0.80-1.93) | 0.341 | 1.37 (0.70-2.70) | 0.360 | 1.30 (1.07-1.57) | 0.009***** | 1.23 (0.92-1.65) | 0.157 |
| **Discussed sex/HIV with**  **parents/guardians** |  |  |  |  |  |  |  |  |
| No | **Reference** |  |  |  |  |  |  |  |
| Yes | 1.20 (0.58-2.47) | 0.622 | 0.86 (0.29-2.61) | 0.793 | 1.20 (0.91-1.59) | 0.192 | 0.74 (0.50-1.09) | 0.127 |
| **Currently using family**  **planning** |  |  |  |  |  |  |  |  |

| No | **Reference** |  |  |  |  |  |  |  |
| --- | --- | --- | --- | --- | --- | --- | --- | --- |
| Yes | 1.20 (0.57-2.51) | 0.632 | 1.48 (0.60-3.65) | 0.394 | 1.75 (1.33-2.31) | <0.001***** | 1.25 (0.85-1.83) | 0.248 |
| **Alcohol use** |  |  |  |  |  |  |  |  |
| No | **Reference** |  |  |  |  |  |  |  |
| Yes | 0.32 (0.06-1.60) | 0.164 | 0.08 (0.01-0.75) | 0.027***** | 0.61 (0.42-0.89) | 0.011***** | 0.46 (0.29-0.73) | 0.001***** |
| **Ever tested for HIV** |  |  |  |  |  |  |  |  |
| No | **Reference** |  |  |  |  |  |  |  |
| Yes | 1.17 (0.73-1.87) | 0.509 | 2.00 (1.03-3.91) | 0.041***** | 0.34 (0.25-0.46) | <0.001***** | 0.67 (0.42-1.08) | 0.100 |
| **PrEP awareness** |  |  |  |  |  |  |  |  |
| No | **Reference** |  |  |  |  |  |  |  |
| Yes | 0.56 (0.20-1.58) | 0.274 | 0.61 (0.20-1.84) | 0.380 | 1.41 (0.91-2.20) | 0.123 | 0.85 (0.48-1.52) | 0.590 |
| **PrEP willingness** |  |  |  |  |  |  |  |  |
| No | **Reference** |  |  |  |  |  |  |  |
| Yes | 0.99 (0.54-1.81) | 0.967 | 0.99 (0.54-1.81) | 0.972 | 1.28 (1.02-1.60) | 0.030***** | 1.24 (0.91-1.69) | 0.180 |
| **PrEP use** |  |  |  |  |  |  |  |  |
| No | **Reference** |  |  |  |  |  |  |  |

| Yes | 0.39 (0.02-7.17) | 0.524 | 0.15 (0.01-2.92) | 0.211 | 3.57 (0.66-19.27) | 0.138 | 4.04 (0.67-24.37) | 0.127 |
| --- | --- | --- | --- | --- | --- | --- | --- | --- |
|  |  |  | F (15,139) =2.67: P=0.0013 | |  |  | F (15,216) =17:17, P<0.001 | |
| ***Abbreviations:*** *PrEP = pre-exposure prophylaxis,* AORs = *Adjusted odds ratios,* CORs = *Crude odds Ratios, CI=* confidence interval, *Statistical significance*  *was defined as* P *< 0.05* | | | | | | | | |

1 **Discussion**
2 This study utilized data from the PHIA-THIS 2022/2023 survey to examine the prevalence and
3 factors associated with RSB among AGYW in the era of PrEP provision. The findings showed
4 that higher RSB was associated with not being in a union among AGYW. HIV testing increased
5 odds of engagement in RSB only among adolescent girls aged 15-17, whereas alcohol use was
6 a protective factor among AGYW. Overall, this study found that RSB among Tanzanian
7 AGYW remains high, with an overall prevalence of 54.1% and a markedly higher burden
8 among adolescent girls aged 15-17 years (59.6%) than among young women aged 18-24 years
9 (53.2%). This level is broadly consistent with evidence that AGYW in SSA carry a substantial
10 RSB burden, especially in settings with high HIV and STI incidence (35). The RSB prevalence
11 observed in the current study is lower than that in Zambia (71.1%) but higher than the school-
12 based prevalence among students from Ethiopia (17.2%) and Rwanda (41%) (17,19,20). These
13 findings suggest that Tanzanian AGYW share the wider regional pattern of persistent sexual
14 vulnerability.
15 Moreover, the increasing prevalence of RSB among adolescent girls aged 15-17 years in the
16 era of PrEP availability shows that there are missed opportunities when it comes to educating
17 about SRH, as the current study and previous studies in Tanzania report that most initiate sex
18 during this time (22,23,25). In Tanzania, communication about SRH issues at the family and
19 community levels is limited, mostly due to cultural norms and embarrassment that discourage
20 parents from engaging in open discussion with adolescents; they often communicate in fear or
21 warning, so that the teens do not learn anything (35,36). Previous studies have identified peers
22 and schools as the main sources of SRH information for adolescents; however, the current
23 school curriculum is often insufficient to address their questions about sexuality, relationships,
24 HIV, and pregnancy, leading many to rely on inaccurate or incomplete information from peers
25 and other informal sources(37). These findings highlight the need to strengthen school-based
26 sexuality education, parent-adolescent communication, and youth-friendly health services to
27 provide accurate information before risky behaviors are established.
28 The socio-demographic findings show that union status was the only factor associated with
29 RSB in both age groups, whereas education level, employment status, wealth index, and
30 residential location were not, a pattern that differs from prior SSA reports documenting
31 associations (17,38). The current study reports that AGYW not in a union have far higher odds
32 of RSB than those in a union, consistent with prior studies conducted in Zambia, Ethiopia, and
33 Rwanda (17,19,20). The increase in RSB among AGYW who are not in a union could be since
34 many are young and in the exploratory stage of sexual activity. As reported in previous studies,
35 they tend to engage with multiple or non-regular sexual partnerships, inconsistent condom use,
36 and transactional sex, which may increase their vulnerability to RSB (17,20,22,31). The latter
37 findings among AGYW who were not in a union may reflect the greater sexual autonomy and
38 relationship transitions experienced during adolescence and young adulthood, a period often
39 characterized by exploration and experimentation with sexual relationships. Acknowledging
40 that both risk- and health-related behavior develop during these stages, shaping behavior and
41 health over the life course. This finding underscores the need for early SRH education and
42 behavior-support services that equip unmarried AGYW with the knowledge and skills to make
43 informed SRH decisions.
44 The protective association between alcohol use and RSB observed in this study contrasts with
45 previous findings from Eastern Africa (19,20,23,38,39). One explanation for this finding is the
46 low prevalence of self-reported alcohol use among AGYW in the survey. This may reflect the
47 impact of recent government policies and public awareness campaigns aimed at restricting
48 alcohol sales to individuals younger than 18 years, which could have reduced alcohol
49 consumption among adolescents. Additionally, the differences in findings may reflect
50 differences in how alcohol use was measured, with the survey capturing any alcohol use rather
51 than hazardous drinking, as well as increased exposure of AGYW who consume alcohol to
52 HIV prevention interventions, including PrEP counseling and risk-reduction services. This
53 finding highlights that HIV prevention programmes should continue incorporating alcohol risk
54 assessment and tailored behavioral interventions for AGYW despite the observed inverse
55 association.
56 In the current study, the majority of AGYW have tested for HIV; this can also be due to the
57 rapid availability of HIV testing initiatives, but also since AGYW who perceive themselves to
58 be at higher risk are more likely to seek HIV testing. However, HIV testing was not a significant
59 predictor of RSB among young women aged 18-24 years but was associated with higher odds
60 of engaging in RSB among adolescent girls aged 15-17 years, consistent with findings from
61 prior studies (40–42). It might be that after testing, some adolescents may perceive a negative
62 HIV test result as reassurance for HIV protection and subsequently underestimate their future
63 HIV risk by engaging in RSB. The latter finding indicates a missed opportunity within current
64 HIV testing services to strengthen comprehensive HIV prevention in the country, as HIV
65 testing among adolescents aged 15-17 should serve as an important entry point for
66 comprehensive HIV prevention. This finding underscores the need to strengthen HIV testing
67 services by integrating post-test risk-reduction counseling, PrEP education, condom
68 promotion, and adolescent-friendly SRH services, while reinforcing that a negative HIV test
69 reflects their current status rather than future protection against HIV.
70 The current findings show that condom non-use remains a dominant component of SRB, with
71 89.0% reporting no condom use at last sex and 85.8% reporting no condom use with a non-
72 marital partner, which is like prior studies conducted in Tanzania (23,43,44). Additionally,
73 other behavioral factors, including health facility visits in the past 12 months, discussions about
74 sex or HIV with a parent or guardian, and current family planning use, were not independently
75 associated with RSB after adjustment, different from previous studies (25,33,43,44). At the
76 same time, the differences likely reflect variation in study populations, age-stratified modeling
77 approach to compare factors among those aged 15-17 and 18-24 years, the focus on sexually
78 active AGYW, who responded to PrEP-related indicators, and differences in how RSB was
79 defined or in the indicators measured in studies.
80 This study reported that PrEP awareness (5.9%), willingness to use PrEP (35.7%), and current
81 PrEP use (1.0%) were low among Tanzanian AGYW; these findings are consistent with
82 previous studies (45–48). The latter result was reported at the time of rapid availability of HIV
83 funding, with the recent 2026 UNAIDS global update indicating that global PrEP uptake has
84 dropped by 38% between 2024 and 2025 in 62 countries(5). After adjustment, none of the
85 PrEP-related factors remained independently associated with RSB. However, this finding is
86 consistent with previous studies from Kenya, Uganda, Botswana, and Senegal, as well as a
87 systematic review carried out among different populations rather than AGYW, which reported
88 little or no evidence that PrEP use independently increases RSB when provided alongside
89 comprehensive HIV prevention services, including counseling and regular follow-up (56–58).
90 This finding is surprising, as PrEP eligibility is based on the same behavioral risk factors
91 assessed in this study, while actual PrEP uptake is influenced by barriers beyond individual
92 risk (49–55). Therefore, further qualitative, longitudinal, and implementation research focusing
93 specifically on AGYW is needed to determine whether these findings are sustained in this
94 priority population.
95 The current study emphasized the importance of rethinking the conceptualization of RSB in
96 the era of effective biomedical HIV prevention.
97 The traditional definitions of RSB were developed before the rapid availability of antiretroviral
98 therapy for HIV prevention, such as PrEP, when condomless sex was directly linked to HIV
99 acquisition risk (1). But as PrEP and treatment as prevention (U=U) become more widely
100 available, the relationship between sexual behavior and HIV risk is a more complex one. For
101 people who are adherent to PrEP or whose partner has sustained viral load suppression, the risk
102 of acquiring HIV may not be as great with condomless sex, but it is still important for
103 preventing STIs, unintended pregnancy, and other adverse SRH outcomes. Consequently, there
104 is growing recognition that traditional definitions of RSB may no longer adequately capture
105 HIV risk and should be reconceptualized to reflect the contemporary prevention landscape
106 Instead of looking at sexual behavior as inherently risky, we need to look at risk in the wider
107 context of biomedical protection, adherence to PrEP, partner viral suppression, relationships,
108 reproductive intentions, and access to integrated SRH services. Future research should
109 therefore develop and validate context-specific measures of sexual risk that reflect the realities
110 of the PrEP era, especially among women at high risk of HIV acquisition.

# 111 Limitations and strengths

112 A major strength is that it used nationally representative data from the Tanzania HIV Impact
113 Survey (THIS) 2022-2023, making the findings generalizable to sexually active adolescent
114 girls and young women in Tanzania. To our knowledge, this is the first nationally
115 representative study in Tanzania to examine RSB in the context of PrEP availability, thereby
116 providing important evidence for HIV prevention policy and programme implementation.
117 However, the cross-sectional design limits causal inference and prevents determination of
118 temporal order between RSB and associated factors. Second, RSB indicators were self-reported
119 and involved sensitive behaviors, so social desirability and recall bias may have led to
120 underreporting of condomless sex, alcohol use, and other stigmatized practices. Third,
121 comparisons across studies should be interpreted cautiously because RSB definitions vary
122 widely by setting, with some studies including early debut, transactional sex, or condom use
123 constructs differently. Fourth, some influential drivers identified elsewhere, including peer
124 pressure, domestic violence, parental neglect, and stigma, were not measured in the dataset, so
125 they could not be explored in the current analysis. Finally, this study used the traditional
126 definition of RSB, which is commonly used in HIV prevention research (1). A more suitable
127 definition in the era of effective biomedical prevention, especially among AGYW, could not
128 be identified.

# 129 Conclusion

130 Risky sexual behavior remains common among Tanzanian adolescent girls and young women,
131 particularly among those aged 15-17 years. Non-union relationship, HIV testing status alcohol
132 use were the main factors associated with risky sexual behavior, highlighting the need for age-
133 specific, youth-friendly sexual and reproductive health interventions that strengthen condom
134 use, delay sexual debut, and improve access to integrated SRH and HIV prevention services,
135 including PrEP, in the early years of adolescents’ lives, enabling them to make the right choices
136 in their sexual relationships and health.

# 137 Data access and ethical approval

138 The study involves secondary analysis of de-identified data. Access to the data used in this
139 analysis was obtained from the PHIA-ICAP Columbia University website after submitting a
140 data analysis plan and a formal request for geospatial data. The PHIA surveys received ethical
141 approval from international and local human subject institutional review boards, including the
142 U.S. Centres for Disease Control and Prevention (CDC); national ethics committees such as
143 the National Institute for Medical Research (NIMR) in Tanzania, and institutional review
144 boards at ICAP at Columbia University or the University of Maryland, Baltimore. The PHIA
145 surveys were funded by the U.S. President’s Emergency Plan for AIDS Relief (PEPFAR), with
146 technical assistance provided by the CDC under cooperative agreements #U2GGH002173 and
147 #U2GGH002172.

# 148 Consent to participate

149 In the original PHIA surveys, all participants provided written informed consent before
150 participation; for minors aged 15-17 years, written assent was obtained in addition to parental
151 or guardian permission.

# 152 Consent for publication

153 Not applicable

# 154 Declaration of conflicting interests

155 The author(s) declared no known financial conflicts of interest with respect to the research,
156 authorship, and/or publication that could have influenced the work reported in this paper.

# 157 Funding statement

158 The Ministry of Education, Science and Technology, Tanzania (MoEST) funded this study
159 through the Higher Education for Economic Transformation (HEET) project (Grant No.
160 P166415). The grant was awarded to Muhimbili University of Health and Allied Sciences
161 (MUHAS), which served as the implementing institution. The content is solely the
162 responsibility of the authors and does not necessarily reflect the official views of the funder.
163 The funder had no role in the study design, data collection and analysis, decision to publish, or
164 preparation of the manuscript.

# 165 Data availability

166 Data from the Tanzania HIV Impact Survey (THIS/PHIA) are publicly available, and
167 researchers may request access by submitting a brief data analysis plan and study objectives
168 through the PHIA data portal: <https://phia-data.icap.columbia.edu/>

# 169 Authors’ contributions

170 MTM was responsible for conceptualization, data curation, formal analysis, funding
171 acquisition, investigation, methodology, project administration, validation, visualization,
172 writing - original draft, and writing - review and editing. DK was involved in supervision and
173 validation. JG contributed to data curation; formal analysis; investigation; methodology,
174 supervision, validation, and writing - review and editing. BG participated in data curation,
175 formal analysis, investigation, methodology, supervision, validation, and writing - review and
176 editing.

# 177 Acknowledgements

178 The authors would like to acknowledge the training contributions of the Structured Population
179 Health Policy and Health Services Research Education (SPHeRE), which is funded by the
180 Health Research Board (HRB) in Ireland**.** The six-month foundation and methodological
181 training provided by the programme were extremely valuable in carrying out this work.

# References

| 182 |  |
| --- | --- |
| 183 | 1. |
| 184 |  |
| 185 |  |
| 186 |  |
| 187 | 2. |
| 188 |  |
| 189 |  |
| 190 | 3. |
| 191 |  |
| 192 |  |
| 193 |  |
| 194 | 4. |
| 195 |  |
| 196 |  |
| 197 | 5. |
| 198 |  |
| 199 | 6. |
| 200 |  |
| 201 | 7. |
| 202 |  |
| 203 |  |
| 204 | 8. |
| 205 |  |

Brawner BM, Alexander KA, Fannin EF, Baker JL, Davis ZM. The Role of Sexual Health Professionals in Developing a Shared Concept of Risky Sexual Behavior and HIV Transmission. Public Health Nurs. 2016 Mar;33(2):139–50. doi:10.1111/phn.12216 PubMed PMID: 26184496; PubMed Central PMCID: PMC4715781.

Unitaid, UK Foreign, Commonwealth & Development Office, Gates Foundation. 2025 HIV Market Report: The state of HIV treatment, testing, and prevention in low- and middle-income countries. 2025 Nov.

TACAIDS TC for A. National HIV Prevention Road Map 2023/24- 2026/27 [Internet]. Mwanza; 2024. Available from: chrome- extension://efaidnbmnnnibpcajpcglclefindmkaj/https:/[/www.tacaids.go.tz/uploads/docum](http://www.tacaids.go.tz/uploads/docum) ents/en-1735984508-Approved%20HIV_prevention_road_map_1.pdf

THIS. PHIA Project [Internet]. 2023 [cited 2024 Jan 11]. Tanzania HIV Impact Survey Summary Sheet 2022-2023. Available from: https://phia.icap.columbia.edu/tanzania- summary-sheet-2022-3/

UNAIDS, Joint United Nations Programme on HIV/AIDS. Global AIDS brief: United to End AIDS. Geneva; 2026. Report CC BY-NC-SA 3.0 IGO.

THIS_Final.pdf [Internet]. [cited 2023 Oct 25]. Available from: https://phia.icap.columbia.edu/wp-content/uploads/2020/02/THIS_Final.pdf TACAIDS TC for A. Tanzania HIV Impact Survey 2022-2023: Final Report [Report] [Internet]. Tanzania; 2023 [cited 2025 Jan 12]. Available from: https://phia.icap.columbia.edu/this-2022-2023-final-report or [http://www.nbs.go.tz](http://www.nbs.go.tz/) UNAIDS_FactSheet_en.pdf [Internet]. [cited 2023 Oct 23]. Available from: https:/[/www.unaids.org/sites/default/files/media_asset/UNAIDS_FactSheet_en.pdf](http://www.unaids.org/sites/default/files/media_asset/UNAIDS_FactSheet_en.pdf)

206 9. Fact Sheet: Young people and HIV and AIDS: Taking action to realize the 90-90-90
207 targets.
208 10. UNAIDS. WOMEN AND HIV A SPOTLIGHT ON ADOLESCENT GIRLS AND
209 YOUNG WOMEN [Internet]. 2019. Available from:
210 https:/[/www.unaids.org/sites/default/files/media_asset/2019_women-and-hiv_en.pdf](http://www.unaids.org/sites/default/files/media_asset/2019_women-and-hiv_en.pdf)
211 11. Morris J, Rushwan H. Adolescent sexual and reproductive health: The global challenges.
212 International Journal of Gynecology & Obstetrics. 2015 Oct 1;131.
213 doi:10.1016/j.ijgo.2015.02.006
214 12. UN Women, Pullen K, Damji N, Kudravtseva E, Guhathakurta A, Stainsby G, et al.
215 INVESTING IN ADOLESCENT GIRLS’ AND YOUNG WOMEN’S LEADERSHIP
216 AND VOICE IN THE HIV RESPONSE CASE STUDIES AND EXPERIENCES
217 [Internet]. 2023. Available from: chrome-
218 extension://efaidnbmnnnibpcajpcglclefindmkaj/https://open.unwomen.org/sites/default/fil
219 es/2024-04/investing_in_young_womens_leadership_hiv.pdf?utm_source=chatgpt.com
220 13. UNICEF. Insights on HIV and Sexual & Reproductive Health Care for Adolescents and
221 Young People. 2017.
222 14. UNFPA T. UNFPA Tanzania [Internet]. 2025 [cited 2025 Nov 9]. Empowering Women,
223 Shaping the Future in Tanzania: A Call for Leadership, Rights, and Equality. Available
224 from: https://tanzania.unfpa.org/en/news/empowering-women-shaping-future-tanzania-
225 call-leadership-rights-and-equality
226 15. Dellar R, Dlamini S, Karim Q. Adolescent girls and young women: key populations for
227 HIV epidemic control. Journal of the International AIDS Society. 2015 Feb 1;18.
228 doi:10.7448/ias.18.2.19408
229 16. Murewanhema G, Musuka G, Moyo P, Moyo E, Dzinamarira T. HIV and adolescent girls
230 and young women in sub‐Saharan Africa: A call for expedited action to reduce new
231 infections. IJID Regions. 2022 Aug 1;5:30–2. doi: 10.1016/j.ijregi.2022.08.009
232 17. Sserwanja Q, Mwamba D, Poon P, Kim J. Prevalence and Factors Associated with Risky
233 Sexual Behaviors Among Sexually Active Female Adolescents in Zambia. Archives of
234 Sexual Behavior. 2022 Aug 29; 52:205–15. doi:10.1007/s10508-022-02385-6
235 18. Matovu J, Bukenya J, Kasozi D, Kisaka S, Kisa R, Nyabigambo A, et al. Sexual-risk
236 behaviours and HIV and syphilis prevalence among in- and out-of-school adolescent girls
237 and young women in Uganda: A cross-sectional study. PLoS ONE. 2021 Sep 10;16. doi:
238 10.1371/journal.pone.0257321
239 19. Srahbzu M, Tirfeneh E. Risky Sexual Behavior and Associated Factors among
240 Adolescents Aged 15-19 Years at Governmental High Schools in Aksum Town, Tigray,
241 Ethiopia, 2019: An Institution-Based, Cross-Sectional Study. BioMed Research
242 International. 2020 Aug 21;2020. doi:10.1155/2020/3719845
243 20. Ndagijimana E, Biracyaza E, Nzayirambaho M. Risky sexual behaviors and their
244 associated factors within high school students from Collège Saint André in Kigali,
245 Rwanda: An institution-based cross-sectional study. Frontiers in Reproductive Health.
246 2023 Mar 3;5. doi:10.3389/frph.2023.1029465
247 21. Odimegwu C, Ugwu N. A multilevel mixed effect analysis of neighbourhood and
248 individual level determinants of risky sexual behaviour among young people in South
249 Africa. Reproductive Health. 2022 May 12;19. doi:10.1186/s12978-022-01407-9
250 22. Rucinski K, Mbita G, Atkins K, Majani E, Komba A, Casalini C, et al. Transactional sex
251 and age-disparate sexual partnerships among adolescent girls and young women in
252 Tanzania. Frontiers in Reproductive Health. 2024 Jul 11;6.
253 doi:10.3389/frph.2024.1360339
254 23. Mcharo R, Olomi W, Mayaud P, Msuya S. Risky sexual behaviours among young adults
255 attending Higher Learning Institutions in Mbeya, Tanzania: implications for STIs and
256 HIV preventive programs. AAS Open Research. 2020 Aug 27;3.
257 doi:10.12688/aasopenres.13123.2
258 24. Mmbaga EJ, Leonard F, Leyna GH. Incidence and Predictors of Adolescent’s Early
259 Sexual Debut after Three Decades of HIV Interventions in Tanzania: A Time to Debut
260 Analysis. PLOS ONE. 2012 Jul 27;7(7): e41700. doi: 10.1371/journal.pone.0041700
261 25. Millanzi W, Osaki K, Kibusi S. Attitude and prevalence of early sexual debut and
262 associated risk sexual behavior among adolescents in Tanzania; Evidence from baseline
263 data in a Randomized Controlled Trial. BMC Public Health. 2023 Sep 9;23.
264 doi:10.1186/s12889-023-16623-6
265 26. Grov C, Westmoreland DA, D’Angelo AB, Pantalone DW. How Has HIV Pre-Exposure
266 Prophylaxis (PrEP) Changed Sex? A Review of Research in a New Era of Bio-behavioral
267 HIV Prevention. The Journal of Sex Research. 2021 Sep 2;58(7):891–913.
268 doi:10.1080/00224499.2021.1936440 PubMed PMID: 34180743.
269 27. Eakle R, Weatherburn P, Bourne A. Understanding user perspectives of and preferences
270 for oral PrEP for HIV prevention in the context of intervention scale-up: a synthesis of
271 evidence from sub-Saharan Africa. Journal of the International AIDS Society.
272 2019;22(S4): e25306. doi:10.1002/jia2.25306
273 28. Quaife M, MacGregor L, Ong JJ, Gafos M, Torres-Rueda S, Grant H, et al. Risk
274 compensation and STI incidence in PrEP programmes. The Lancet HIV. 2020 Apr 1;7(4):
275 e222–3. doi:10.1016/S2352-3018(19)30333-9
276 29. Rojas Castro D, Delabre RM, Molina J. Give PrEP a chance: moving on from the “risk
277 compensation” concept. J Int AIDS Soc. 2019 Aug 30;22(Suppl Suppl 6): e25351.
278 doi:10.1002/jia2.25351 PubMed PMID: 31468693; PubMed Central PMCID:
279 PMC6715948.
280 30. Han H, Murray S, Mbita G, Bangser M, Rucinski K, Komba A, et al. Characterizing a
281 sexual health and HIV risk stratification scale for sexually active adolescent girls and
282 young women (AGYW) in Tanzania. PLoS ONE. 2021 Mar 18;16. doi:
283 10.1371/journal.pone.0248153
284 31. Millanzi W. Sexual behaviours, self-reported health outcomes, and associated
285 determinants among adolescents: a cross-sectional survey in Tanzania. International
286 Journal of Adolescence and Youth. 2025 Aug 6;30. doi:10.1080/02673843.2025.2543933
287 32. Bago M, Stephano E, Kessy SA, Tibenderana J, Majengo VG, Oguma ED, et al.
288 Determinants of comprehensive HIV and AIDS knowledge among Tanzanian adolescent
289 girls and young women: a multilevel application of Andersen’s behavioral model based
290 on national survey. Frontiers in Public Health. 2025 Sep 9;13.
291 doi:10.3389/fpubh.2025.1643422
292 33. Stephano E, Bago M, Tian Y, Ruhighira J, Mtoro M, Nyundo A, et al. Factors associated
293 with sexual behaviours among adolescents in a rural Tanzania: evidence from the African
294 Research, Implementation Science, and Education (ARISE) Network. Discover Public
295 Health. 2025 May 22;22. doi:10.1186/s12982-025-00678-4
296 34. Eliufoo E, Mtoro M, Godfrey V, Bago M, Kessy I, Millanzi W, et al. Prevalence and
297 associated factors of early sexual initiation among female youth in Tanzania: a
298 nationwide survey. BMC Public Health. 2025 Feb 28;25. doi:10.1186/s12889-025-
299 21533-w
300 35. Kajula LJ, Sheon N, De Vries H, Kaaya SF, Aarø LE. Dynamics of parent-adolescent
301 communication on sexual health and HIV/AIDS in Tanzania. AIDS Behav. 2014 Jan;18
302 Suppl 1: S69-74. doi:10.1007/s10461-013-0634-6 PubMed PMID: 24101099.
303 36. Millanzi WC, Osaki KM, Kibusi SM. Parent-adolescent communication about sexual and
304 reproductive health and its determinants among adolescents: Baseline findings from a
305 Randomized Controlled Trial in Tanzania. Sage Open. 2023 Oct
306 1;13(4):21582440231216281. doi:10.1177/21582440231216281
307 37. Mcharo RD, Mayaud P, Msuya SE. Where and how do young people like to get their
308 sexual and reproductive health (SRH) information? Experiences from students in higher
309 learning institutions in Mbeya, Tanzania: a cross-sectional study. BMC Public Health.
310 2021 Sep 16;21(1):1683. doi:10.1186/s12889-021-11728-2 PubMed PMID: 34530796;
311 PubMed Central PMCID: PMC8444543.
312 38. Workneh B, Zegeye A, Tamir T, Mekonen E. Risky sexual behavior and associated
313 factors among reproductive age women in eastern African countries: a multilevel analysis
314 of the recent demographic and health survey. BMC Women’s Health. 2024 Apr 30;24.
315 doi:10.1186/s12905-024-03107-x
316 39. Wagle A, Karki K. Prevalence of risky sexual behaviour and its associated factors among
317 youths of Pokhara metropolitan city, Nepal: a cross-sectional study. BMJ Public Health.
318 2024 Oct 1;2. doi:10.1136/bmjph-2024-000945
319 40. Pachena A, Musekiwa A. Trends in HIV Testing and Associated Factors among
320 Adolescent Girls and Young Women in Zimbabwe: Cross-Sectional Analysis of
321 Demographic and Health Survey Data from 2005 to 2015. International Journal of
322 Environmental Research and Public Health. 2022 Jan;19(9):5165.
323 doi:10.3390/ijerph19095165
324 41. Okeke-Obayemi D, Alawode O, Okeke S, Mwaniki S, Mabhala A, Mullens A, et al.
325 Prevalence and determinants of HIV testing among adolescent girls and young women in
326 28 sub-Saharan African countries. BMC Public Health. 2025 Nov 29;26.
327 doi:10.1186/s12889-025-25828-w
328 42. Musekiwa A, Bamogo A, Shisana O, Robsky K, Zuma K, Zungu NP, et al. Prevalence of
329 self-reported HIV testing and associated factors among adolescent girls and young
330 women in South Africa: Results from a 2017 nationally representative population-based
331 HIV survey. Public Health Pract (Oxf). 2021 Feb 6; 2:100093. doi:
332 10.1016/j.puhip.2021.100093 PubMed PMID: 36101581; PubMed Central PMCID:
333 PMC9461291.
334 43. Ndlovu R, Moyo PL. Prevalence and correlates of risky sexual behaviour among in-
335 school adolescents in Bulawayo, Zimbabwe: a cross-sectional study. BMJ Public Health.
336 2026 Jan 1;4. doi:10.1136/bmjph-2025-004364
337 44. Ndlovu R, Moyo PL. Exploring prevalence of risky sexual behaviour among adolescents
338 in high schools: A systematic review. SAGE Open Medicine. 2026 Feb 1;14.
339 doi:10.1177/20503121251413045
340 45. Kapola AJ, Musoke R, Manayon GM, Fussi HF, Bakari HM, Ally HM, et al.
341 Pre‑exposure Prophylaxis Awareness and Endorsement among Adolescents and Young
342 Adults in Tanzania: Insights from the 2022 Demographic and Health Survey. Annals of
343 Global Health. 2025 Jan 24;91(1). doi:10.5334/aogh.4589
344 46. Kampamba M, Nelumbu NN, Hikaambo CN, Mudenda S, Hangoma JM, Kunda M, et al.
345 Awareness and willingness to use pre‐exposure prophylaxis among the University of
346 Zambia students: A cross‐sectional study. Health Science Reports. 2024.
347 doi:10.1002/HSR2.70060
348 47. Lubogo P, Namirembe P. Brief communication: Pre-exposure prophylaxis utilization and
349 associated factors among sexually active adolescents and young adults in Soroti city,
350 Uganda. AIDS Research and Therapy. 2025. doi:10.1186/S12981-025-00832-7
351 48. Agwang W, Nangendo J, Nabikande S, Okello TR, Tusabe J, Semitala FC, et al. Factors
352 associated with willingness to take Pre-Exposure Prophylaxis (PrEP) among high-risk
353 adolescent boys and young men in Masese fishing community, Uganda. PLOS Global
354 Public Health. 2023. doi: 10.1371/JOURNAL.PGPH.0001191
355 49. Mwakilasa MT, Mwijage A, Mushy S, Amour M, Sirili N, Maziku E, et al. “They are not
356 HIV treatments drugs; they are preventive drugs (PrEP)”. Experiences of PrEP uptake
357 among vulnerable adolescent girls and young women in Tanzania. PLOS ONE. 2025 Jan
358 8;20. doi: 10.1371/journal.pone.0313501
359 50. Admassu M, Nöstlinger C, Hensen B. Barriers to PrEP use and adherence among
360 adolescent girls and young women in Eastern, Southern, and Western Africa: a scoping
361 review. BMC Women’s Health. 2024 Dec 1;24. doi:10.1186/s12905-024-03516-y
362 51. Chen-Charles J, Davey DJ, Toska E, Seeley J, Bekker LG. PrEP Uptake and Utilisation
363 Among Adolescent Girls and Young Women in Sub-Saharan Africa: A Scoping Review.
364 AIDS and Behavior. 2025 Feb 28; 29:1876–96. doi:10.1007/s10461-025-04656-4
365 52. Pilgrim N, Jani N, Mathur S, Kahabuka C, Saria V, Makyao N, et al. Provider
366 perspectives on PrEP for adolescent girls and young women in Tanzania: The role of
367 provider biases and quality of care. PLoS ONE. 2018 Apr 27;13. doi:
368 10.1371/journal.pone.0196280
369 53. Stangl A, Bryan C, Barré I, Ramskin L, Lees S, Ndimande-Khoza N, et al. The influence
370 of stigma on PrEP uptake among adolescent girls and young women in Johannesburg,
371 South Africa and Mwanza, Tanzania: Qualitative findings from the EMPOWER study.
372 PLOS One. 2026 Feb 3;21. doi: 10.1371/journal.pone.0339380
373 54. Trisha Ramraj, Witness Chirinda, Kim Jonas, Darshini Govindasamy, Ngcwalisa
374 Amanda Jama, Tracy McClinton Appollis, et al. Service delivery models that promote
375 linkages to PrEP for adolescent girls and young women and men in sub-Saharan Africa: a
376 scoping review. 2023. doi:10.1136/bmjopen-2022-061503
377 55. Kayesu I, Mayanja Y, Nakirijja C, Machira YW, Price M, Seeley J, et al. Uptake of and
378 adherence to oral pre-exposure prophylaxis among adolescent girls and young women at
379 high risk of HIV-infection in Kampala, Uganda: a qualitative study of experiences,
380 facilitators and barriers. BMC Women’s Health. 2022;22(440) :(10 November 2022).
381 Located at: CABI Databases. doi:10.1186/s12905-022-02018-z
382 56. Traeger MW, Schroeder SE, Wright EJ, Hellard ME, Cornelisse VJ, Doyle JS, et al.
383 Effects of Pre-exposure Prophylaxis for the Prevention of Human Immunodeficiency
384 Virus Infection on Sexual Risk Behavior in Men Who Have Sex with Men: A Systematic
385 Review and Meta-analysis. Clin Infect Dis. 2018 Sep 1;67(5):676–86.
386 doi:10.1093/cid/ciy182
387 57. Ortblad KF, Stalter RM, Bukusi EA, Ngure K, Mujugura A, Celum C, et al. No Evidence
388 of Sexual Risk Compensation Following PrEP Initiation Among Heterosexual HIV
389 Serodiscordant Couples in Kenya and Uganda. AIDS Behav. 2020 May;24(5):1365–75.
390 doi:10.1007/s10461-019-02720-4 PubMed PMID: 31696370; PubMed Central PMCID:
391 PMC7156350.
392 58. Mugwanya KK, Donnell D, Celum C, Thomas KK, Ndase P, Mugo N, et al. Sexual
393 behaviour of heterosexual men and women receiving antiretroviral pre-exposure
394 prophylaxis for HIV prevention: a longitudinal analysis. The Lancet Infectious Diseases.

395

396

397

398

2013 Dec 1;13(12):1021–8. doi:10.1016/S1473-3099(13)70226-3

Figure	[Click here to access/download;Figure;20260716 Figure 1.tif](https://www2.cloud.editorialmanager.com/pgph/download.aspx?id=848364&guid=f21b4c8a-f15a-40c5-af51-81a1dc3b89e7&scheme=1)

Total number of respondent surveyed in the 2022/2023

33366

. 23759 excluded because age is above	24 years.

F

lr

9904

**r**

- 4242 exclude all male respondent

5662

l

- 90 removed HIV status positive

5572

.	2090 removed because they were not sexual

.

active

d

Total number of sexually active AGYW aged 15-24

**3482**	-	years included in the analysis

Figure	[Click here to access/download;Figure;20260716 Figure 2.tif](https://www2.cloud.editorialmanager.com/pgph/download.aspx?id=848365&guid=1b78ebb0-713f-48d4-8702-366014f6e734&scheme=1)

I	**Independent variables**	)	I	**Dependent variables**	)

**Socio-demographic factors**

- Respondent age
- Education level
- Wealth status
- Employment status (12 months)
- Union status
- Residencial areas	f
- Geographical zones

**Risk sexual behaviour**

- Age at first sexual intercourse below 15

**Behavioural and sexual health factors**	year old

- Family planning use	• Age at first marriage
- Accessed health services (12 months)	before 15 years old
- Ever talked about sex/HIV with	• Number of sexual

parent/guardian

.-	partners in the past

- Ever tested for HIV	^12^ months
- Alcohol use (12 months)	• Engaging in sexual
- Condom use at last sexual encounter	intercourse without a (past 12 months)	condom with a

non-marital or

non-cohabiting partner

PrEP awareness PrEP willingness PrEP use

Figure	[Click here to access/download;Figure;20260716 Figure 3.tif](https://www2.cloud.editorialmanager.com/pgph/download.aspx?id=848366&guid=2493e30b-82b7-4c2c-be91-c0a9e9a45e08&scheme=1)[<https://www2.cloud.editorialmanager.com/pgph/download.aspx?id=848366&guid=2493e30b-82b7-4c2c-be91-c0a9e9a45e08&scheme=1>](https://www2.cloud.editorialmanager.com/pgph/download.aspx?id=848366&guid=2493e30b-82b7-4c2c-be91-c0a9e9a45e08&scheme=1)

Supporting Information

[Click here to access/download](https://www2.cloud.editorialmanager.com/pgph/download.aspx?id=848363&guid=23b1b59a-da8d-4fe6-b32a-4ba3efa39c4b&scheme=1)

[**Supporting Information** Plos GPH Supporting file 1.docx](https://www2.cloud.editorialmanager.com/pgph/download.aspx?id=848363&guid=23b1b59a-da8d-4fe6-b32a-4ba3efa39c4b&scheme=1)
