# Supplementary material for: Risky sexual behaviors and associated factors among adolescent girls and young women in the context of HIV prevention pre-exposure prophylaxis in Tanzania: Analysis of the Tanzania HIV Impact Survey 2022–2023
Source: PLOS Glob Public Health. 2026 Oct 23;6(10):e0007311. doi: 10.1371/journal.pgph.0007311 (PMC13693934; doi:10.1371/journal.pgph.0007311)
Supplement: Supplementary file 7 [file pgph.0007311.s010.docx]

**Author Feedback to editor and reviewer Comments**

Dear Editorial Team,

Thank you for your time and consideration of our manuscript, **Risky Sexual Behaviors and Associated Factors among Adolescent Girls and Young Women in the Context of HIV Prevention Pre-exposure Prophylaxis in Tanzania: Analysis of the Tanzania HIV Impact Survey 2022-2023***”* for publication in PLOS Global Public Health

We are grateful for the insightful feedback provided by the reviewers. We have carefully revised the manuscript in response to their comments and addressed each point in a point-by-point manner below. We believe that the revisions have strengthened the manuscript and improved its clarity.

We hope that the updated version meets the expectations of the editors and reviewers, and we remain grateful for the constructive feedback that helped refine this work.

1. Please upload all main figures as separate Figure files in .tif or .eps format only and remove the embedded figures from the manuscript file.
For more information about how to convert and format your figure files please see our guidelines:<https://journals.plos.org/globalpublichealth/s/figures>

**Author response:** The figure has been updated based on the journal’s requirements.

2.      Please ensure that the Title in your manuscript file and the Title provided in your online submission form are the same.

**Author response:** The title has been changed to match the one uploaded online.

3.     Before we proceed, we’ll require some additional clarification to ensure your submission adheres to the PLOS ONE Data Availability policy (<https://journals.plos.org/plosone/s/data-availability>).
Please note that the data we ask authors to share is not necessarily the full and/or raw data set collected in the course of their research, but rather the "minimal data set", which we define as consisting of the data set used to reach the conclusions drawn in the manuscript with related metadata and methods, and any additional data required to replicate the reported study findings in their entirety. 
Depending on the nature of your study, this can include any or all of the following:
- The values behind the means, standard deviations and other measures reported;
- The values used to build graphs;
- The points extracted from images for analysis.
For studies involving third-party data (i.e., data not owned or collected by the authors), we encourage authors to share any data specific to their analyses that they can legally distribute. PLOS recognizes, however, that authors may be using third-party data they do not have the rights to share. When third-party data cannot be publicly shared, authors must provide all information necessary for interested researchers to apply to gain access to the data in the text of the Data Availability statement. This statement will be published online alongside your article should it be accepted for publication.

For any third-party data that the authors cannot legally distribute, please provide the following information in an updated and complete Data Availability statement:

a) Please kindly explain in detail how others can access the minimal datasets you used in this study from the third-party websites you accessed. Please provide any relevant information necessary to gain immediate access to the specific minimal datasets used in your work (e.g. contact information, specific URLs, dataset titles, etc.).

b) Please clarify in detail whether interested researchers can replicate your study findings in their entirety by directly obtaining the data from the third party sources in your Data Availability statement and following the information outlined in your Methods section.

c) Please also confirm whether the authors had any special access privileges that others would not have when attempting to access the minimal data you used from these third party sources.

Alternatively, if there are no restrictions, please upload the complete minimal anonymized data set necessary to replicate your study findings to a stable, public repository and provide us with the relevant URLs, DOIs, or accession numbers.

For a list of recommended repositories, please see: <https://journals.plos.org/plosone/s/recommended-repositories>

You also have the option of uploading the data as Supporting Information files, but we would recommend depositing data directly to a data repository if possible.

We will update your Data Availability statement on your behalf to reflect the information you provide.

**Author response:** The data availability statement has been updated based on the provided recommendation.
